# PhyloMissForest: a random forest framework to construct phylogenetic trees with missing data

**DOI:** 10.1186/s12864-022-08540-6

**Published:** 2022-05-18

**Authors:** Diogo Pinheiro, Sergio Santander-Jimenéz, Aleksandar Ilic

**Affiliations:** 1 INESC-ID, Instituto Superior Técnico, Universidade de Lisboa, Rua Alves Redol 9, Lisboa, 1000-029 Portugal; 2 Department of Computer and Communications Technologies, University of Extremadura, Campus universitario s/n, Cáceres, 10003 Spain

**Keywords:** Phylogenetic tree, Missing data imputation, Machine learning, Random forest

## Abstract

**Background:**

In the pursuit of a better understanding of biodiversity, evolutionary biologists rely on the study of phylogenetic relationships to illustrate the course of evolution. The relationships among natural organisms, depicted in the shape of phylogenetic trees, not only help to understand evolutionary history but also have a wide range of additional applications in science. One of the most challenging problems that arise when building phylogenetic trees is the presence of missing biological data. More specifically, the possibility of inferring wrong phylogenetic trees increases proportionally to the amount of missing values in the input data. Although there are methods proposed to deal with this issue, their applicability and accuracy is often restricted by different constraints.

**Results:**

We propose a framework, called PhyloMissForest, to impute missing entries in phylogenetic distance matrices and infer accurate evolutionary relationships. PhyloMissForest is built upon a random forest structure that infers the missing entries of the input data, based on the known parts of it. PhyloMissForest contributes with a robust and configurable framework that incorporates multiple search strategies and machine learning, complemented by phylogenetic techniques, to provide a more accurate inference of lost phylogenetic distances. We evaluate our framework by examining three real-world datasets, two DNA-based sequence alignments and one containing amino acid data, and two additional instances with simulated DNA data. Moreover, we follow a design of experiments methodology to define the hyperparameter values of our algorithm, which is a concise method, preferable in comparison to the well-known exhaustive parameters search. By varying the percentages of missing data from 5% to 60%, we generally outperform the state-of-the-art alternative imputation techniques in the tests conducted on real DNA data. In addition, significant improvements in execution time are observed for the amino acid instance. The results observed on simulated data also denote the attainment of improved imputations when dealing with large percentages of missing data.

**Conclusions:**

By merging multiple search strategies, machine learning, and phylogenetic techniques, PhyloMissForest provides a highly customizable and robust framework for phylogenetic missing data imputation, with significant topological accuracy and effective speedups over the state of the art.

**Supplementary Information:**

The online version contains supplementary material available at (10.1186/s12864-022-08540-6).

## Background

The understanding of the evolutionary history and relationships among individuals or groups has become a key research topic over the years. Phylogenetic studies represent a fundamental tool not only in the evolutionary biology field but also in a wide range of other important application domains [1]. Phylogenetic insights help conservation researchers to study and make decisions about the protection of endangered species and conservation policies [2, 3]. The application of phylogenetics also revealed to be important in forensic sciences [4]. It also improves the investigation of pathogens in molecular epidemiology, thus being a useful tool to understand and fight against infectious diseases [5, 6]. In fact, phylogenetic analyses are actively contributing to the identification and characterization of SARS-CoV-2 lineages, as shown in [7, 8]. The inclusion of phylogenetics in medicine proved to be a tool that significantly improves the detection of complex diseases like cancer [9, 10]. In the area of pharmacology, the analysis of phylogenetic trees also helps researchers in drug development in a variety of ways [11].

In order to describe evolutionary hypotheses, a graphical structure called phylogenetic tree is built. A phylogenetic tree *T*=(*V*,*E*) illustrates evolutionary relationships among the organisms characterized in the node set *V*, through the definition of linkages in the branch set *E*. The internal nodes in *V* are known as Hypothetical Taxonomic Units (HTUs), since they denote potential ancestors whose evolution resulted in the Operational Taxonomic Units (OTUs) located in the terminal nodes. The methodology used to infer phylogenetic trees is divided into two major groups: character-based methods and distance-based methods [12]. While the former uses a multiple sequence alignment to generate a set of possible phylogenetic trees directly from the sequences at hand, the latter processes the given sequences to construct a phylogenetic pairwise distance matrix instead and then infer a phylogenetic tree from the matrix. The most popular character-based methods are: maximum parsimony, maximum likelihood, and Bayesian methods [1]. As for distance-based methods, the ones that are mostly used are: unweighted pairwise group method with arithmetic means (UPGMA), weighted pairwise group method with arithmetic means (WPGMA), neighbor joining (NJ) and Fitch-Margoliash (FM) [13].

When analysing large-scale datasets, character-based methods, in particular maximum likelihood and Bayesian methods, tend to be computationally demanding. In contrast, distance based methods typically provide a less demanding approach to infer phylogenies, which is crucial to overcome strict time constraints in phylogenetic analyses and also to define reliable starting points in complex biological scenarios [14]. Moreover, under certain circumstances, distance-based methods have shown competitive and faster results when comparing with character-based methods [15–18]. Therefore, distance-based methods will be the main point of focus in this research work.

Distance-based methods follow two major steps: 1) compute the distance matrix and 2) obtain the phylogenetic tree from the distance matrix. In order to perform the second step, the pairwise distance matrix obtained in the first step is typically expected to be complete, i.e, all its values should be known. However, the presence of missing data can lead to failure in the straightforward calculation of phylogenetic distances, thus making the process of building a phylogenetic tree a challenging task. In fact, the probability of missing data increases with the amount of data to be analysed [19]. Missing data in the phylogenetics field may occur for several reasons: failure of experimental work [14], data generation protocols, approaches to taxon and gene sampling and gene birth and loss [20]. Missing data can also appear due to the lack of biological material, imprecision of experimental methods and for a combination of unpredictable reasons [21].

With the aim of building the pairwise distance matrix, the first step to be performed is the acquisition of the sequences. Afterwards, the second step is the alignment of the sequences. Finished the process of sequence alignment, the dissimilarity between each sequence is then calculated, i.e., when comparing two sequences, each column is analysed and the distance between them is the number of different columns they share. The calculated dissimilarity is later converted into evolutionary distances by using correction factors derived from a substitution model and the result is the pairwise distance matrix constructed from the alignment of the sequences. Hence, the problem of missing data in phylogenetic distance matrices arises from the missing characters in the sequence alignment [22].

**Fig. 1:**
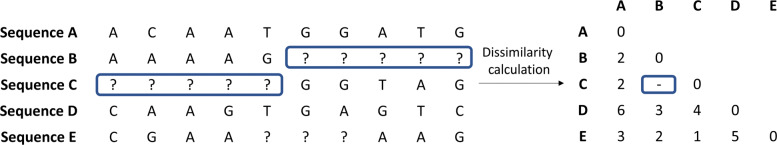
Missing matrix inputs obtained from incomplete sequences

In Fig. 1, there is an example of a dissimilarity matrix obtained from an alignment with missing characters. In order to calculate the distance between two sequences, after being aligned, they have to share known subsequences between each other. In the example illustrated in Fig. 1, it can be observed that the distance between sequence *B* and *C* cannot be directly calculated, since the known subsequences do not overlap. That is, in the part sequence *B* has known characters, sequence *C* has missing characters, represented with the question mark, and vice versa. On the other hand, the distances between sequence *E* and other sequences are not affected by this issue, in spite of also having unknown values. In this case, the columns in which *E* has an unknown value and the OTU it is compared to has a known value do not count towards the distance.

Different studies revealed that, under the presence of missing data, the possibility of inferring wrong phylogenetic trees significantly increases [23, 24]. Therefore, there is a need for efficient estimation techniques to deal with missing data in phylogenetic pairwise distance matrices. The approaches existing in the literature can be classified into two major groups: direct and indirect methods. The direct methods infer the phylogenetic tree directly from the partial distance matrix, while the indirect methods first implement an imputation strategy to fill the missing information in the distance matrix and, after that, construct the phylogenetic tree from the imputed matrix.

State-of-the-art direct methods rely on different techniques to infer the phylogenetic tree directly from the incomplete distance matrix, such as the triangles method [25], a least-squares (LS) method named MW-modified [21], and an adaptation of NJ to deal with incomplete distance matrices [26]. The applicability of the referred methodologies depends on a combination of restrictions that need to be satisfied. For example, in order to add a new element to a tree in the triangles method, it must share at least two known distances with elements within the tree. On the other hand, MW-modified requires that the distances are additive, that is, the distances in the matrix must correspond to the distances in the phylogenetic tree. Distances are said to be additive when they satisfy the four-point metric condition, i.e. the sum of the distances *d*_*AB*_+*d*_*CD*_ is ≤ max(*d*_*AC*_+*d*_*BD*_,*d*_*AD*_+*d*_*BC*_) for any four OTUs *A*, *B*, *C*, *D*. When this condition is verified, distances can be fitted so that the branch lengths in the path between a pair of OTUs equal the genetic distance between them. Such combinations of restrictions therefore impose limitations on the application of the existent direct approaches.

Several studies proposed different approaches to define indirect methods, where the inference of the missing distances is first tackled to proceed afterwards with the construction of the phylogenetic tree from the imputed distance matrix. Among these proposals, it can be highlighted a heuristic approach named LASSO [14], an LS-based approach with multivariate optimization called DAMBE [22], a statistical method called SIA [27], and two Machine Learning (ML) techniques: matrix factorization and autoencoder [28]. In order to apply LASSO, the molecular clock hypothesis needs to be assumed, meaning that sequence divergence must accumulate over time at a constant rate. This assumption establishes that genetic distances are linearly proportional to the time elapsed, leading to phylogenies that satisfy the ultrametricity property. Ultrametric trees are rooted trees in which each leaf has the same distance to the root. However, this constraint is difficult to be ensured in real-world data, since evolutionary rates are dependent on multiple factors, such as mutation rates, generation times, or population sizes. DAMBE proved that it is possible to build a phylogenetic tree without assuming the molecular clock hypothesis. Nevertheless, this method cannot ensure the phylogenetic reconstruction with large percentages of missing data, which is also the main issue with the SIA method. The matrix factorization and autoencoder approaches from [28] represent the current state-of-the-art strategies for indirect phylogenetic imputation, yet an in-depth parameter tuning and the customization of the underlying architectures in these methods are still important questions to be addressed.

Given the issues identified in the current state-of-the-art methodologies, the definition of efficient algorithmic strategies for dealing with missing data in phylogenetic distance matrices remains an open, challenging problem. Additionally, there is a demand for robust solutions that can be configured and adapted to different situations according to the features of phylogenetic datasets with missing data.

In order to address the challenges that arise when inferring phylogenies in the presence of missing data, we introduce an ML-based framework designated as PhyloMissForest. The devised approach is built upon random forest based unsupervised imputation algorithms that are combined with a variety of search techniques, coupled with phylogenetic techniques and criteria, to accurately conduct imputations over phylogenetic pairwise distance matrices. In this way, PhyloMissForest encapsulates, in a single customizable framework, a set of different algorithmic strategies guided by phylogenetic criteria to effectively address the missing data imputation problem in real-world phylogenetic scenarios.

## Results

This section undertakes the experimental evaluation of the proposed PhyloMissForest framework, reporting and analysing the attained results. The experimental methodology followed these steps: i) for an input distance matrix derived from sequence data, missing entries were randomly introduced until accomplishing the desired percentage of missing data (from 5% to 60%); ii) we applied PhyloMissForest to perform the imputation and recover the missing entries; iii) the phylogenetic tree recovered from the matrix generated by PhyloMissForest was compared with a ground-truth tree, which was derived from the original distance matrix. In this way, the success of the framework to recover the original data and, consequently, the underlying phylogenetic relationships can be measured. The distance-based method used to infer the referred phylogenetic trees was NJ. Since this algorithm returns an unrooted bifurcating phylogenetic tree, in order to compare the tree obtained via imputation with the one constructed from the original matrix without missing data, the Robinson-Foulds (RF) metric was adopted as evaluation criterion [29].

RF compares two phylogenetic trees by inspecting differences at the splits induced by the edges of the trees. Given two unrooted phylogenetic trees A and B, the RF metric calculates the number of edges in A or B that are not in both trees, being each edge identified by the bipartition it induces on the leaf set [30]. In order to get the percentage of the difference between A and B, the normalised Robinson-Foulds (NRF) metric is used. This metric is calculated as the ratio between the RF score and the maximum possible number of splits i.e. the maximum possible RF. Since the maximum possible RF, obtained by a pair of bifurcating trees, is 2*N*−6, where *N* is the number of OTUs, the NRF expression is given as follows:

1 $$ NRF=\frac{RF}{2N-6}   $$

By multiplying the value of the NRF by 100, the percentage of error that the imputation introduces is obtained. If the NRF is 0%, the reconstruction of the phylogenetic tree is accurate and the topology of the imputed solution matches the reference one.

Across the experimental evaluation, we evaluate the performance of PhyloMissForest by making comparisons with the two techniques that represent the state of the art in ML-based phylogenetic imputation: matrix factorization and autoencoder [28]. For this purpose, three different real-world datasets were employed in the experiments: 1) a dataset with 9 sequences of baculovirus data [28, 31]; 2) an amino acid dataset with 37 sequences of *xylona heveae* fungi [32]; and 3) a DNA dataset with 55 sequences of green plants [33]. Two simulated datasets, with 40 [34] and 201 [35] DNA sequences respectively, were also considered in the experimentation to broaden the spectrum of problem sizes under analysis. The original distance matrices were generated from the sequence data contained in these datasets with the exception of the baculovirus matrix, which was obtained from https://github.com/Ananya-Bhattacharjee/ImputeDistances.

The following statistical methodology was adopted to examine differences in the NRF samples reported by the compared methods [36]. First, Kolmogorov-Smirnov tests were conducted to detect if the samples followed a Gaussian distribution. If so, Levene tests were then used to analyse homogeneity in variances. In case of detecting Gaussian-distributed samples with homogeneous variances, ANOVA was applied to analyse statistical significance. In any other case, Wilcoxon-Mann-Whitney was used instead. Due to the number of samples and the variability observed in the evaluated scenarios, a confidence level of 90% was considered in this analysis.

The herein presented experimental evaluation has been performed in a multicore Intel i9-10980XE CPU, running at 3.20 GHz with 128GB (8 ×16GB) of DDR4 RAM. The operating system is Linux, with a compiler GCC version 7.3.0. The programming language adopted to develop the framework is Python, version 3.7.7.

### Comparison with ML-based state-of-the-art methods

Throughout this section, we test both bootstrap and non-bootstrap configurations of PhyloMissForest and compare them with two key state-of-the-art approaches: autoencoder and matrix factorization [28]. In order to perform this comparative evaluation, we analyse the different datasets considered in this work with percentages of missing data from 5% to 60%, with increments of 5%. For each percentage of missing data, 10 distance matrices are tested. Taking into account experimental constraints and the effect of stochastic components, each matrix is executed 5 times and the average of the 5 runs is calculated, accounting for a total of 50 runs per missing data percentage. We perform this procedure in our algorithm (both configurations) and autoencoder. In the case of matrix factorization, the restrictions imposed by the huge execution time of this approach limited the experimentation to 10 runs per missing data percentage. The results of each percentage of missing data are the average of the 10 tested matrices.

### 9 ×9 dataset

**Fig. 2:**
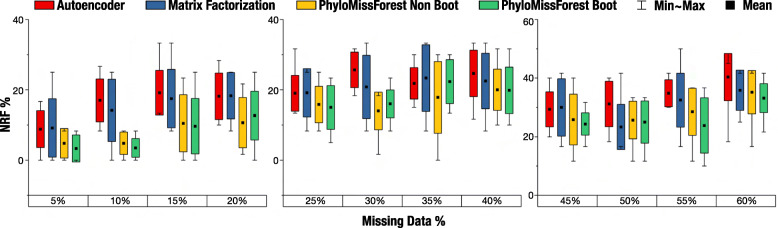
Results obtained with the 9 ×9 dataset. Box plots with the mean, min (best), max (worst) and standard deviation of NRF

For the 9 ×9 dataset, Fig. 2 depicts, for each percentage of missing data tested, the NRF results obtained by our approach and the two ML state-of-the-art approaches. Since our main goal is to minimize the value of NRF, lower values in Fig. 2 denote better results. Table 1 reports the results of the statistical analysis of NRF samples.

**Table 1:** Statistical testing of NRF results for the 9x9 dataset, with regard to autoencoder (AE) and matrix factorization (MF). Statistically significant improvements achieved by PhyloMissForest (under any of the considered configuration profiles) are denoted as $\checkmark $, while non-significant differences are marked with ×

Dataset	%Missing	Non-bootstrap *p*-values		Bootstrap *p*-values		PhyloMissForest diff.	
		Vs. AE	Vs. MF	Vs. AE	Vs. MF	Vs. AE	Vs. MF
9x9	5%	**0.09**	0.25	**0.02**	**0.10**	$\checkmark $	$\checkmark $
	10%	**0.00**	**0.02**	**0.00**	**0.01**	$\checkmark $	$\checkmark $
	15%	**0.02**	**0.08**	**0.01**	**0.09**	$\checkmark $	$\checkmark $
	20%	**0.02**	**0.02**	0.11	0.14	$\checkmark $	$\checkmark $
	25%	0.25	0.28	0.22	0.12	×	×
	30%	**0.00**	0.14	**0.00**	0.12	$\checkmark $	×
	35%	0.58	0.17	0.97	0.44	×	×
	40%	**0.09**	0.31	0.19	0.35	$\checkmark $	×
	45%	0.31	0.44	**0.06**	**0.10**	$\checkmark $	$\checkmark $
	50%	0.14	0.17	**0.09**	0.53	$\checkmark $	×
	55%	0.11	0.44	**0.01**	**0.09**	$\checkmark $	$\checkmark $
	60%	**0.01**	0.91	**0.00**	0.35	$\checkmark $	×

Bold values refer to *p*-values denoting statistically significant improvements

Focusing first on the comparison between our approach and autoencoder, it can be observed that PhyloMissForest leads to general improvements over the autoencoder approach, achieving a lower (better) mean NRF value in most of the percentages of missing data considered in this dataset. More specifically, the non-bootstrap configuration recovers the targeted phylogenetic tree more accurately than autoencoder for each percentage of missing data under study. Regarding the bootstrap case, it can be observed that the method under this configuration also improves autoencoder under different missing data percentages. The only scenario where the autoencoder approach is slightly better than the bootstrap configuration is for 35% of missing data. Nevertheless, when considering both configurations, PhyloMissForest provides overall NRF improvements over autoencoder, since our approach supports the two combinations of parameters in accordance with the configuration profile selected by the user. According to Table 1, these improvements are statistically significant in ten out of twelve cases i.e. 83.3%.

The performance obtained with PhyloMissForest is also better when compared to the matrix factorization method. It can be observed in Fig. 2 that our approach attains better mean NRF values for almost all percentages of missing data, with the exception of the scenarios with 50% of missing entries. From a statistical perspective, significant improvements are achieved in half of the evaluated scenarios, being the differences more noticeable within missing data ranges between 5% and 20%.

Apart from the comparisons described above, another interesting aspect is the comparison between our two combinations of parameters (bootstrap and non-bootstrap profiles). As shown in Fig. 2, the bootstrap configuration reports the best results in nine out of the twelve percentages of missing data herein tested. The minimum, maximum and standard deviation obtained in each percentage of missing data are also important information to analyse. As denoted in the box plots of Fig. 2, our framework reaches 0% of NRF, which is the desirable goal, in at least one matrix for the percentages between 5% and 20%, while the other methods only manage to achieve this percentage in the matrices with 5% and 10% of missing data. Hence, the results herein achieved suggest that PhyloMissForest has better capabilities to recover the full topology of the phylogenetic trees, since it was able to reach 0% of NRF with up to 20% of missing data, while the current state-of-art methodologies only satisfactorily handled at most percentages of 10%.

### 37 ×37 dataset

**Fig. 3:**
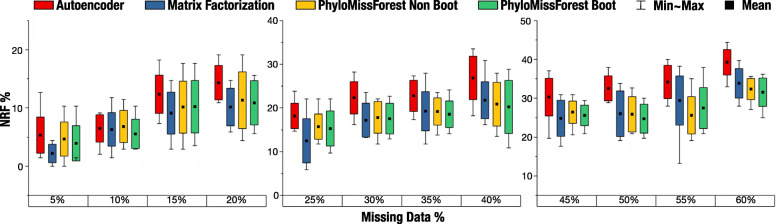
Results obtained with the 37 ×37 dataset. Box plots with the mean, min (best), max (worst) and standard deviation of NRF

Figure 3 presents the results obtained for the dataset with 37 OTUs. Again, we tested percentages of missing data between 5% and 60%, with increments of 5%. Table 2 introduces the results of the statistical tests performed for this dataset.

**Table 2:** Statistical testing of NRF results for the 37x37 dataset, with regard to autoencoder (AE) and matrix factorization (MF). Statistically significant improvements achieved by PhyloMissForest (under any of the considered configuration profiles) are denoted as $\checkmark $, while non-significant differences are marked with ×

Dataset	%Missing	Non-bootstrap *p*-values		Bootstrap *p*-values		PhyloMissForest diff.	
		Vs. AE	Vs. MF	Vs. AE	Vs. MF	Vs. AE	Vs. MF
37x37	5%	0.63	0.04	0.22	0.17	×	×
	10%	0.97	0.80	0.35	0.53	×	×
	15%	0.28	0.63	0.31	0.44	×	×
	20%	0.17	0.63	**0.10**	0.58	$\checkmark $	×
	25%	**0.08**	0.11	**0.06**	0.17	$\checkmark $	×
	30%	**0.02**	0.74	**0.02**	0.91	$\checkmark $	×
	35%	**0.04**	0.85	**0.02**	0.68	$\checkmark $	×
	40%	**0.01**	0.74	**0.03**	0.68	$\checkmark $	×
	45%	**0.02**	0.63	**0.01**	0.91	$\checkmark $	×
	50%	**0.01**	0.91	**0.00**	0.68	$\checkmark $	×
	55%	**0.00**	1.00	**0.01**	0.39	$\checkmark $	×
	60%	**0.00**	0.48	**0.00**	0.28	$\checkmark $	×

Bold values refer to *p*-values denoting statistically significant improvements

When considering the two configurations of parameters jointly, the proposed PhyloMissForest framework obtains a lower (better) value of the mean NRF than the state-of-the-art autoencoder method for every percentage of missing data. When the two configuration profiles are separately considered, it can be concluded that PhyloMissForest with bootstrap achieves better average NRF results than autoencoder for all tested percentages of missing data, with statistically significant differences in the missing data intervals from 20% to 60%. On the other hand, the non-bootstrap configuration statistically outperforms autoencoder in 66.7% of the evaluated scenarios. In this sense, although autoencoder provides marginally better solutions (0.5%) than the non-bootstrap profile in the matrices with 10% of missing data, these differences are reported to be non-significant from a statistical perspective (*p*-value = 0.97).

When compared with the matrix factorization method, the proposed approach attains better NRF averages for six out of twelve tested scenarios. Nevertheless, the statistical testing of NRF samples between PhyloMissForest and matrix factorization reveals that both methods show comparable performance i.e. non-significant differences in this dataset. It is worth noting that the 37 ×37 dataset contains amino acid data, which turns the data imputation problem more challenging when compared with a DNA dataset, due to the different number of possible characters in the alignment. In such complex scenarios, the observed execution times give account of the benefits of applying PhyloMissForest with regard to matrix factorization. Particularly, the matrix factorization approach requires 17 minutes in this dataset, while PhyloMissForest is able to successfully finish the imputation process in only 25 seconds under the non-bootstrap profile or 7 minutes when bootstrapping is enabled. Therefore, effective speedups can be observed when applying the proposed framework in this context. A more detailed analysis of execution time is addressed in the Results discussion section.

When comparing the non-bootstrap and bootstrap configuration profiles supported by PhyloMissForest, it is verified that the bootstrap approach achieves improved results ten out of twelve times. Therefore, by enabling bootstrapping, the accuracy of PhyloMissForest is boosted in 83.3% of the cases, in comparison to the configuration profile that does not involve bootstrap techniques.

### 55 ×55 dataset

**Fig. 4:**
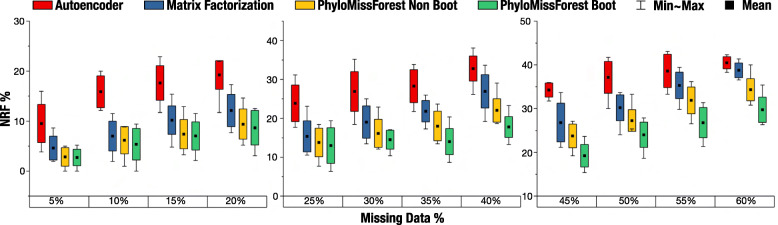
Results obtained with the 55 ×55 dataset. Box plots with the mean, min (best), max (worst) and standard deviation of NRF

Similarly to the 9 ×9 and 37 ×37 datasets, Fig. 4 presents the results obtained by PhyloMissForest and the state-of-the-art methods in the dataset with 55 OTUs. The statistical evaluation of NRF samples is provided in Table 3.

**Table 3:** Statistical testing of NRF results for the 55x55 dataset, with regard to autoencoder (AE) and matrix factorization (MF). Statistically significant improvements achieved by PhyloMissForest (under any of the considered configuration profiles) are denoted as $\checkmark $, while non-significant differences are marked with ×

Dataset	%Missing	Non-bootstrap *p*-values		Bootstrap *p*-values		PhyloMissForest diff.	
		Vs. AE	Vs. MF	Vs. AE	Vs. MF	Vs. AE	Vs. MF
55x55	5%	**0.00**	0.17	**0.00**	**0.10**	$\checkmark $	$\checkmark $
	10%	**0.00**	0.58	**0.00**	0.25	$\checkmark $	×
	15%	**0.00**	**0.05**	**0.00**	**0.02**	$\checkmark $	$\checkmark $
	20%	**0.00**	0.12	**0.00**	**0.09**	$\checkmark $	$\checkmark $
	25%	**0.00**	0.48	**0.00**	0.25	$\checkmark $	×
	30%	**0.00**	**0.09**	**0.00**	**0.02**	$\checkmark $	$\checkmark $
	35%	**0.00**	**0.03**	**0.00**	**0.00**	$\checkmark $	$\checkmark $
	40%	**0.00**	**0.03**	**0.00**	**0.00**	$\checkmark $	$\checkmark $
	45%	**0.00**	**0.09**	**0.00**	**0.00**	$\checkmark $	$\checkmark $
	50%	**0.00**	**0.02**	**0.00**	**0.00**	$\checkmark $	$\checkmark $
	55%	**0.00**	**0.03**	**0.00**	**0.00**	$\checkmark $	$\checkmark $
	60%	**0.00**	**0.00**	**0.00**	**0.00**	$\checkmark $	$\checkmark $

Bold values refer to *p*-values denoting statistically significant improvements

Starting with the comparison between our approach and autoencoder, our results denote that PhyloMissForest reports better solutions in all the twelve percentages of missing data herein examined. In fact, such improvements are verified regardless of the configuration profile (bootstrap or non-bootstrap) adopted in the framework. It is worth highlighting that, for this particular dataset in all the percentages of missing data, the improvement observed in the average NRF score is always more than 6.8%, thus representing a significant difference in performance between our approach and the state-of-the-art autoencoder. This idea is supported by the output of the statistical analysis, which confirms the attainment of statistically significant improvements in all the cases under study.

When comparing our approach with the matrix factorization method, it can be observed that PhyloMissForest also achieves improved NRF performance over the competing approach in overall terms. According to Table 3, statistically significant improvements are observed in 83% of the tested scenarios. These results therefore suggest the relevance of the framework in this dataset, especially when large percentages of missing data are considered. In these difficult scenarios, any configuration profile of PhyloMissForest is able to outperform the matrix factorization method, i.e., better results are attained independently of the profile set in our algorithm. As for the comparison between the bootstrap and non-bootstrap cases, more satisfying results are observed when bootstrap strategies are adopted.

By examining the minimum values of NRF, it can be concluded that only the proposed PhyloMissForest is able to recover 0% of NRF in at least one matrix. Additionally, the maximum NRF value obtained in each percentage of missing data is always lower for our approach than for the state-of-the-art techniques.

### Simulated datasets

**Fig. 5:**
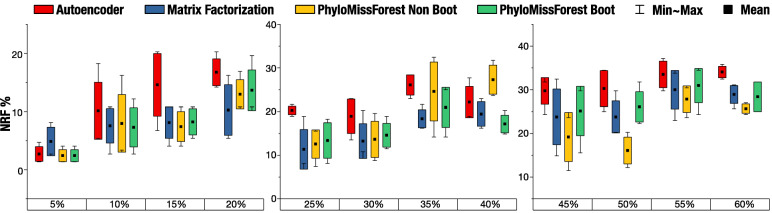
Results obtained with the 40 ×40 dataset. Box plots with the mean, min (best), max (worst) and standard deviation of NRF

The evaluation of PhyloMissForest in synthetic scenarios is undertaken next. Figure 5 and Table 4 report the NRF results obtained by the proposed framework for two simulated datasets (40x40 and 201x201). Table 5 illustrates the statistical assessment of the results attained in these datasets, in comparison to autoencoder and matrix factorization. Due to the increased execution times associated to the processing of the 201x201 dataset, the evaluation of this problem instance involved missing data percentages between 10% and 20%.

**Table 4:** Mean NRF results (%) and standard deviations for the 201x201 dataset. N/A refers to situations where matrix factorization did not finish execution in an experimental time window of 48 hours

Dataset	%Missing	PhyloMissForest		AutoEncoder	Matrix Factorization
		Non-bootstrap	Bootstrap		
201x201	10%	15.20 ±2	**14.70 ±2**	18.81 ±3	N/A
	15%	19.02 ±1	**18.89 ±2**	23.64 ±2	N/A
	20%	**21.16 ±1**	21.26 ±1	25.08 ±1	N/A

Bold values refer the best NRF results in the comparison

**Table 5:** Statistical testing of NRF results for the 40x40 and 201x201 datasets, with regard to autoencoder (AE) and matrix factorization (MF). Statistically significant improvements achieved by PhyloMissForest (under any of the considered configuration profiles) are denoted as $\checkmark $, while non-significant differences are marked with ×. N/A refers to situations where matrix factorization did not finish execution in an experimental time window of 48 hours

Dataset	%Missing	Non-bootstrap *p*-values		Bootstrap *p*-values		PhyloMissForest diff.	
		Vs. AE	Vs. MF	Vs. AE	Vs. MF	Vs. AE	Vs. MF
40x40	5%	0.69	**0.05**	0.69	**0.05**	×	$\checkmark $
	10%	0.31	0.69	0.42	0.42	×	×
	15%	**0.05**	0.69	**0.05**	1.00	$\checkmark $	×
	20%	**0.05**	0.31	**0.10**	0.22	$\checkmark $	×
	25%	**0.01**	0.69	**0.01**	0.55	$\checkmark $	×
	30%	**0.10**	1.00	**0.10**	0.31	$\checkmark $	×
	35%	0.84	0.15	**0.05**	0.31	$\checkmark $	×
	40%	0.05	0.01	**0.03**	**0.05**	$\checkmark $	$\checkmark $
	45%	**0.01**	**0.05**	**0.10**	0.55	$\checkmark $	$\checkmark $
	50%	**0.01**	**0.01**	0.15	0.22	$\checkmark $	$\checkmark $
	55%	**0.10**	0.84	0.84	0.42	$\checkmark $	×
	60%	**0.01**	**0.03**	**0.01**	0.84	$\checkmark $	$\checkmark $
201x201	10%	**0.05**	N/A	**0.05**	N/A	$\checkmark $	N/A
	15%	**0.01**	N/A	**0.01**	N/A	$\checkmark $	N/A
	20%	**0.01**	N/A	**0.01**	N/A	$\checkmark $	N/A

Bold values refer to *p*-values denoting statistically significant improvements

Focusing first on the 40x40 dataset, the comparison with autoencoder in this simulated scenario suggests similar implications to the ones verified in real sequence data. In particular, the proposed approach achieves statistically significant improvements over autoencoder in 83.3% of the cases under study (considering the results from both bootstrap and non-bootstrap configuration profiles). Regarding the comparison with matrix factorization, the results in this problem size (40x40) imply a compromise between the observations reported for the 37x37 and 55x55 datasets. While PhyloMissForest and matrix factorization tend to show comparable performance in scenarios with low-medium percentages of missing data, the proposed approach is more likely to successfully achieve statistically significant improvements when large percentages of missing data are involved in the imputation process.

The analysis of the 201x201 dataset gives account of the significant behaviour of PhyloMissForest when dealing with larger problem sizes. Statistically significant improvements over autoencoder are reported in all the evaluated scenarios. As for matrix factorization, the execution times of this tool surpassed an experimental window of 48 hours per matrix instance, thus not being able to report solutions in the considered time period. In contrast, the proposed PhyloMissForest represents in this sense a more suitable approach to process complex datasets. These results therefore support the relevance of the proposed framework also in the case of synthetic datasets.

### Comparison with other alternative methods

In order to further examine the performance of PhyloMissForest, comparisons with other approaches for missing data imputation are herein presented. Particularly, we have performed comparisons with two popular methods: LASSO [14] and DAMBE [22]. Table 6 introduces the comparison of mean NRF scores on real-world datasets (9x9, 37x37, and 55x55), while Table 7 reports the results observed in simulated datasets (40x40 and 201x201), considering for PhyloMissForest the results achieved by the most accurate configuration profile. These tables also include the *p*-values resulting from the statistical comparison of the results obtained by PhyloMissForest and the alternative techniques, in order to verify the attainment of statistically significant differences.

**Table 6:** Comparisons with LASSO and DAMBE on real-world datasets: mean NRF values and *p*-values obtained in the statistical testing of PhyloMissForest samples over the alternative approaches. Lower NRF values denote better quality. N/A denotes scenarios where DAMBE was not able to find any suitable solution

Dataset	%Missing	NRF scores			*p*-values	
		PhyloMissForest	LASSO	DAMBE	vs. LASSO	vs. DAMBE
9x9	5%	**3.33**	9.17	23.61	**0.00**	**0.00**
	10%	**3.50**	8.33	29.17	**0.00**	**0.00**
	15%	**9.67**	14.17	41.67	0.25	**0.00**
	20%	**10.67**	13.33	38.54	0.35	**0.00**
	25%	**15.00**	16.67	39.58	0.53	**0.01**
	30%	**14.00**	17.50	36.11	0.48	**0.01**
	35%	17.83	**15.00**	N/A	0.44	N/A
	40%	**19.83**	21.67	N/A	0.58	N/A
	45%	24.33	**20.17**	N/A	0.35	N/A
	50%	25.00	**22.83**	N/A	0.63	N/A
	55%	**23.83**	29.17	N/A	0.35	N/A
	60%	33.17	**30.00**	N/A	0.68	N/A
37x37	5%	3.94	7.35	**3.19**	**0.01**	0.79
	10%	**5.56**	7.50	6.72	**0.06**	0.54
	15%	10.15	**9.71**	10.54	0.91	0.96
	20%	10.88	**10.74**	N/A	0.53	N/A
	25%	15.29	**12.50**	N/A	0.11	N/A
	30%	17.56	**15.00**	N/A	0.19	N/A
	35%	18.56	**16.76**	N/A	0.25	N/A
	40%	20.24	**18.24**	N/A	0.48	N/A
	45%	25.59	**21.32**	N/A	0.00	N/A
	50%	24.74	**22.21**	N/A	0.14	N/A
	55%	27.47	**24.12**	N/A	0.63	N/A
	60%	31.53	**30.15**	N/A	0.53	N/A
55x55	5%	**2.73**	20.58	N/A	**0.00**	N/A
	10%	**5.38**	21.63	N/A	**0.00**	N/A
	15%	**7.04**	22.79	N/A	**0.00**	N/A
	20%	**8.67**	22.31	N/A	**0.00**	N/A
	25%	**13.02**	24.90	N/A	**0.00**	N/A
	30%	**14.52**	26.73	N/A	**0.00**	N/A
	35%	**14.02**	27.21	N/A	**0.00**	N/A
	40%	**17.79**	30.38	N/A	**0.00**	N/A
	45%	**19.23**	28.94	N/A	**0.00**	N/A
	50%	**24.02**	33.27	N/A	**0.00**	N/A
	55%	**26.79**	35.10	N/A	**0.00**	N/A
	60%	**29.73**	35.10	N/A	**0.00**	N/A

Bold values in the “NRF scores” columns denote the best NRF scores in the comparison, while in the *p*-values columns they refer to *p*-values denoting statistically significant improvements

**Table 7:** Comparisons with LASSO and DAMBE on simulated datasets: mean NRF values and *p*-values obtained in the statistical testing of PhyloMissForest samples over the alternative approaches. Lower NRF values denote better quality. N/A denotes scenarios where DAMBE was not able to find any suitable solution

Dataset	%Missing	NRF scores			*p*-values	
		PhyloMissForest	LASSO	DAMBE	vs. LASSO	vs. DAMBE
40x40	5%	**2.43**	20.54	N/A	**0.00**	N/A
	10%	**7.30**	21.89	N/A	**0.00**	N/A
	15%	**7.43**	20.00	N/A	**0.00**	N/A
	20%	**12.97**	22.70	N/A	**0.00**	N/A
	25%	**12.57**	23.24	N/A	**0.00**	N/A
	30%	**13.65**	21.35	N/A	**0.02**	N/A
	35%	**20.95**	28.38	N/A	**0.00**	N/A
	40%	**17.16**	24.59	N/A	**0.00**	N/A
	45%	**19.19**	31.89	N/A	**0.00**	N/A
	50%	**16.08**	29.46	N/A	**0.00**	N/A
	55%	**27.84**	32.97	N/A	**0.03**	N/A
	60%	**25.68**	31.89	N/A	**0.00**	N/A
201x201	10%	**14.70**	33.84	N/A	**0.00**	N/A
	15%	**18.89**	35.56	N/A	**0.00**	N/A
	20%	**21.16**	34.85	N/A	**0.00**	N/A

Bold values in the “NRF scores” columns denote the best NRF scores in the comparison, while in the “*p*-values” columns they refer to *p*-values denoting statistically significant improvements

Focusing first on the results obtained on real-world datasets, it can observed that PhyloMissForest and LASSO achieve statistically comparable NRF results (*p*-values ≥ 0.1, with a confidence level of 90%) in most of the tests involving the 9x9 and 37x37 datasets. In these scenarios, statistically significant improvements are reported by PhyloMissForest when dealing with missing data percentages of 5% and 10%, with mean NRF scores of 3.33%–3.50% (for 9x9) and 3.94%–5.56% (for 37x37). On the other hand, the only case where LASSO managed to obtain significant higher accuracy was in the 37x37 dataset with 45% of missing data. Nevertheless, the results obtained in the 55x55 dataset denote that the use of PhyloMissForest leads to noticeable boosting in NRF accuracy with regard to LASSO when a higher number of sequences are involved. In fact, statistically significant improvements (*p*-values around 0.0) are achieved by PhyloMissForest in this dataset for all the considered missing data percentages. An additional advantage of PhyloMissForest over LASSO is given by the fact that the proposed approach is not restricted by the molecular clock assumption, i.e. the reported trees are not forced to be ultrametric as in LASSO. As for DAMBE, it is worth remarking that this method was not able to handle missing data percentages beyond 15% for 37x37 and 30% for 9x9. In the case of the 55x55 dataset, DAMBE did not manage to report solutions in any of the evaluated scenarios.

The results reported in simulated datasets confirm the relevance of the proposed PhyloMissForest with regard to the alternative approaches. More specifically, PhyloMissForest achieved statistically significant improvements over LASSO in all the targeted evaluation scenarios, for both 40x40 and 201x201. In addition, the proposed approach successfully handled all the considered missing data percentages in these datasets, showing better applicability than the DAMBE method. These results denote the practical interest of PhyloMissForest and the performance gains attained by the combination of ML techniques and phylogenetic-aware search strategies available in the proposed framework.

### Phylogenetic trees reconstruction

**Fig. 6:**
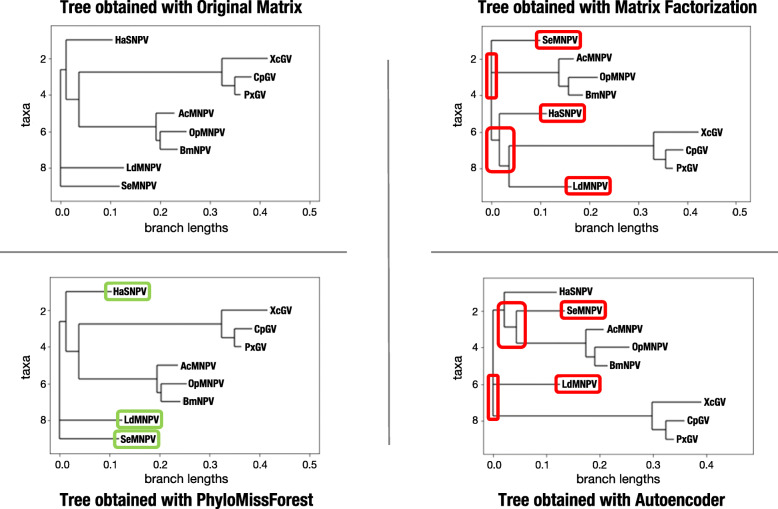
Phylogenetic trees estimated with the full distance matrix on the upper left, in comparison with the trees obtained with PhyloMissForest (bottom left), matrix factorization (upper right) and autoencoder (bottom right) in the 9 ×9 dataset with 5% of missing data

In order to better depict the capabilities of our framework to recover phylogenetic trees with missing data, in comparison to autoencoder and matrix factorization, Fig. 6 illustrates a graphical example of the solutions generated by each method. This example refers to the 9 ×9 dataset with 5% of missing data, showing the best solutions reported by each approach for the first 5% matrix instance considered in the experimentation. We herein highlight the similarities and divergences of the recovered phylogenetic trees in comparison to the phylogenetic topology inferred from the original distance matrix. The main idea is to examine how close the topology outputted by PhyloMissForest (in the presence of missing data) is with regard to the neighbor-joining solution (derived from the full original distances).

From the analysis of Fig. 6, it can be verified that, for both autoencoder and matrix factorization, the recovered phylogenetic trees have noticeable differences from a topological perspective. It can be identified the presence of two main clades: 1) XcGV, CpGV and PxGV; and 2) AcMNPV, OpMNPV and BmNPV. They not only appear in the tree obtained with full information, but also in the trees estimated with the two referred methods. However, the relationships of these groups with the other OTUs were lost in the imputation process implemented in both autoencoder and matrix factorization. This issue results, for both state-of-the-art algorithms, in a significant loss of accuracy in the recovered solutions (with a NRF score of 16.6%).

On the other hand, PhyloMissForest is able to fully recover the topology of the neighbor-joining tree. For both bootstrap and non-bootstrap profiles, the phylogenetic tree is correctly recovered with 0% of NRF. In fact, by examining the branch lengths in this example, it can be observed that the proposed framework is able to lead in this scenario to branch length values that are almost equal to the ones established by the phylogeny obtained with the full matrix (mean error = 0.001). Considering the cumulative branch lengths (i.e. the sum of the branch lengths from the most distant ancestor to the OTU), the most noticeable differences with regard to the competing methods are as follows:

- The cumulative branch lengths estimated by matrix factorization for the OTUs AcMNPV, OpMNPV, BmNPV, and LdMNPV are 0.17, 0.22, 0.19, and 0.16. In contrast, PhyloMissForest and the reference tree define cumulative branch lengths about 0.22, 0.25, 0.22, and 0.12, respectively.
- The cumulative branch lengths reported by autoencoder for the OTUs XcGV, CpGV, PxGV, and SeMNPV are 0.39, 0.36, 0.34, and 0.13. On the other hand, the reference lengths and the ones obtained by PhyloMissForest are around 0.41, 0.38, 0.36, and 0.11, respectively.

## Discussion

According to the experimental evaluation herein conducted, PhyloMissForest provides an accurate framework to solve the missing data imputation problem in phylogenetic distance matrices. The main idea behind the proposal consists of coupling a random forest based imputation procedure with different pairwise matrix search strategies and phylogenetic evaluation criteria (a detailed description can be found in the Methods section). An important characteristic of the devised approach therefore lies in the flexibility it offers in terms of the imputation strategies and configuration profiles that can be used, in accordance with the characteristics of the input data. Our framework has a wide range of configurable possibilities that can be adopted during the imputation and evaluation cycles, so that the combination of decisions that best fit the data can be selected. We herein present and describe the combination of decisions employed in the experimental section, proceeding afterwards with the hyperparameter tuning study and discussion of the obtained results.

### Framework engine study

With the aim of examining the strategies that better behave in a variety of real-world datasets, the experimental evaluation of PhyloMissForest started by analysing the different matrix search strategies integrated in the imputation cycle of our framework, as well as the accuracy of the supported stop criteria. Herein we present a summary of this analysis and the attained results (to visualize a more detailed report please refer to Additional file 1, provided as supplementary material). In order to properly assess the wide range of possibilities that our framework offers to the user, we divided the study in two main phases:

1. **Component Phase**: During the execution of the random forest imputation cycle, different matrices can be inferred in each iteration (i.e. upper triangular matrix, lower triangular matrix, or the mean between both). In this first phase, we tested the three possible criteria implemented in PhyloMissForest to choose the best distance matrix at each imputation step: LS, minimal evolution (ME) or the variable set difference [37]. Two additional strategies were also studied: turning the matrix symmetric after each column imputation and turning it symmetric only at the end of all columns imputation. As such, five different approaches to choose the best matrix were examined, each one combined with stochastic decisions or Q-matrix based decisions in the process of building each decision tree;
2. **Stop Criterion Phase**: In this second phase, we evaluated the effectiveness of the two stop criteria that our framework supports: the variable set difference and an LS-based stop criterion. For both phases, the initial guess of the missing values is performed by first imputing the average value of each column and then turning the matrix symmetric.

From this study, we concluded that significant improvements in the performance and accuracy of the inference can be achieved when we incorporate the guidance provided by phylogenetic LS in the decisions of the algorithm. In this way, the method is able to identify the matrices that best fit phylogenetic quality criteria. Among the combinations of decisions that obtained the best results, it can be highlighted the configuration that analyses and selects the best matrix using LS. As previously remarked, this approach relies on splitting the non-symmetric matrix derived from the random forest into three candidate symmetrical matrices, aiming to better explore the matrix search space iteratively. This is an important feature that distinguishes our proposal from other imputation approaches oriented towards mixed-type data imputation. Moreover, the referred configuration solves the ties that take place during the decision trees building process by using stochastic decisions and LS as the preferred stop criterion. This combination of decisions and strategies led PhyloMissForest to the most satisfying overall behaviour and was therefore employed to undertake the experimental analyses presented in this work.

In order to illustrate the performance of PhyloMissForest under this imputation scheme (denoted as *Split-LS-Rand*), Table 8 reports the mean NRF scores achieved on the real-world datasets considering different missing data percentages (five matrices per missing data percentage). The results of *Split-LS-Rand* are compared with the best results achievable with any potential configuration of search strategies supported in PhyloMissForest (without hyperparameter tuning), as well as the ones reported by the reference, mixed-type data imputation method MissForest [37]. The proposed approach leads to significant improvements over MissForest in all the evaluated tests, with accumulated NRF scores of 172.8% (*Split-LS-Rand*) vs. 226.3% (MissForest). These results give account of the improved imputation capabilities that PhyloMissForest provides for phylogenetic data.

**Table 8:** Comparisons of NRF values between PhyloMissForest and the baseline algorithm MissForest [37]. “Split-LS-Rand” refers to the configuration where LS is incorporated for guidance purposes in the different steps of PhyloMissForest, while “Best Observed” represents the best results reported with any possible configuration of search strategies. Lower values denote better quality

Dataset	%Missing	PhyloMissForest		Mixed-type
		Split-LS-Rand	Best Observed	MissForest
9x9	5%	4.6	**2.9**	5.0
	10%	7.5	**6.7**	11.7
	15%	**7.5**	**7.5**	12.1
	20%	12.5	**9.2**	13.8
	25%	**12.5**	**12.5**	19.2
	30%	**12.9**	**12.9**	17.9
37x37	5%	**1.2**	**1.2**	2.0
	10%	**2.8**	**2.8**	4.6
	15%	**4.6**	**4.6**	7.0
	20%	6.8	**6.5**	9.4
	25%	**9.3**	**9.3**	14.0
	30%	**13.2**	**13.2**	16.2
55x55	5%	3.4	**3.0**	6.3
	10%	5.9	**5.7**	8.7
	15%	11.0	**10.1**	14.7
	20%	**14.3**	**14.3**	16.9
	25%	20.5	**19.7**	22.0
	30%	**22.3**	**22.3**	25.0

Bold values refer to the best NRF values in the comparison

### Hyperparameter study

The process of testing and defining hyperparameters in ML methods, which is also known as hyperparameters tuning, is one of the most challenging tasks to be performed. A widely adopted technique used for this purpose is grid search, in which the user defines a set of values for each parameter and then an exhaustive test of stochastic combinations is tested. This approach potentially incurs in a large number of runs, turning it inefficient. Aiming to turn this task more methodological, a design of experiments (DOE) was applied in our work. [38] points out that the process of tuning hyperparameters in ML can be enhanced by applying DOE, so this approach was adopted to configure hyperparameters in the PhyloMissForest framework.

PhyloMissForest supports a variety of parameters (which are detailed in the Methods section, more precisely in the Hyperparameters section). Some of them are based on the size of the dataset, while there are others that are not directly related to the dataset in usage. For example, the minimum number of samples that a node has to contain to be considered as a leaf (*Min Leaf*) and the maximum depth each decision tree can grow (*Max Depth*) are defined by considering the size of the dataset in usage. Particularly, the value of these parameters is given by a floating-point number between 0 and 1, which is multiplied by the size of the dataset in order to fit the specific dimensions of the evaluated data. The maximum number of features that are analysed (*Max Features*) can be established in a similar way. Other hyperparameters supported by the proposal include the size of the bootstrapped datasets (*Size of the bootstrap*) and the number of decision trees to be considered in each random forest (*Number of trees*).

The hyperparameter study was conducted under two main profiles: bootstrap = 0, which refers to the non-bootstrap case presented in the experimental evaluation and bootstrap = 1, which refers to the bootstrap case. For each of them, a study composed of three steps was performed: 1) Parameter-by-parameter analysis, aiming to understand reasonable ranges for each parameter value; 2) Factorial DOE to filter which are the three parameters that have the strongest statistical meaning, fixing the values of the other parameters; 3) Box-Behnken design [39] to set the values of the parameters that remained to be defined from the previous step.

This analysis was performed by using the *Statistica* software [40]. From it, it can be concluded that the optimal combination of parameters for the evaluated real-world datasets is the one defined in Table 9. Herein we presented a summary of the hyperparameter study (please refer to Additional file 2, provided as supplementary material, for a more comprehensive review of this study). When dealing with user-specified datasets, these two configuration profiles can serve as starting points to obtain satisfying results. Further enhanced performance can be attained through fine-grained parameter tuning in accordance with the characteristics of the input data.

**Table 9:** Final parameter settings for PhyloMissForest under non-bootstrap and bootstrap profiles

Parameters	Non-Bootstrap	Bootstrap
Bootstrap	0	1
Size of the bootstrap	-	1
Number of trees	30	50
Max Features	0.25	1
Max Depth	1	1
Min Leaf	0.01	0.13

### Results discussion

Using the insights from the framework and hyperparameters studies, the experimental evaluation of PhyloMissForest involved the comparative analysis of the configuration profiles identified in Table 9 with the current state-of-the-art ML competitors: matrix factorization and autoencoder. The experimentation was conducted in three real-world problem instances with sizes between 9 and 55 OTUs and missing data percentages between 5% and 60%, as well as two simulated datasets with 40 and 201 OTUs. In overall terms, for the DNA datasets, our framework attains noticeable improvements over the state-of-the-art methods, especially when addressing larger problem instances and missing data percentages. For the amino acid dataset, PhyloMissForest achieves improved solutions when comparing with autoencoder, whereas in the comparison with matrix factorization our framework is able to reach comparable results in this problem instance.

In order to further highlight the relevance of the attained results, it is important to examine the execution times required by each competing method. The analysis of execution time represents a fundamental tool to decide the most efficient and fitting strategies that can be adopted to impute phylogenetic distances for a given dataset in real-world scenarios. Table 10 presents the mean execution times reported by PhyloMissForest under the non-bootstrap and bootstrap profiles, with regard to the alternative ML approaches autoencoder and matrix factorization.

**Table 10:** Mean execution times obtained with autoencoder, matrix factorization, and PhyloMissForest (non-bootstrap and bootstrap profiles)

Dataset	Autoencoder	Matrix Factorization	Non-Bootstrap	Bootstrap
9 ×9	25s	18s	1s	20s
37 ×37	37s	17min	25s	7min
40 ×40	9min	35min	1.7min	20min
55 ×55	2min	53min	2min	25min
201 ×201	1.5h	>48h	6h	34h

From the results shown in Table 10, it can be concluded that PhyloMissForest, under the non-bootstrap configuration profile, reports the fastest executions in the comparison for the 9 ×9, 37 ×37, and 40 ×40 datasets. When running PhyloMissForest under non-bootstrap on these smaller datasets, our proposal also tends to achieve better results in terms of average NRF scores with regard to the autoencoder approach. Regarding the second configuration profile, when PhyloMissForest is run with bootstrapping enabled, better NRF results can be attained at the expense of a penalty in execution time. In comparison to matrix factorization, the framework under the bootstrap configuration is able to achieve significant reduction in execution time, while also improving the overall accuracy of the imputation in the DNA scenarios. As for the amino acid dataset, the proposed approach was able to achieve comparable success ratio with regard to matrix factorization. However, while the matrix factorization method requires 17 minutes to impute distances in this scenario, our approach with bootstrapping only requires 7 minutes.

When addressing a larger dataset, as in the case of 201 ×201, it can be observed that the proposal requires more execution time than the autoencoder approach when using the configuration profiles suggested in Table 9. It can therefore be highlighted the significant scalability shown by autoencoder as the size of the dataset is increased. In this sense, it is important to emphasize that the flexibility of the PhyloMissForest framework allows the user to define a configuration profile that successfully minimizes the time required to address imputations in larger datasets. For instance, by just reducing the number of trees from 30 to 5, the non-bootstrap profile significantly reduces execution time from 6 to 1.3 hours. Under this configuration, although a small penalty in NRF quality can be observed (0.2%), the proposal is able to go a step further with regard to the relevant results reported by autoencoder, from both topological accuracy and execution time perspectives. As for matrix factorization, the experimentation highlighted the increased time requirements of this reference method for larger problem sizes, in comparison to PhyloMissForest with the non-bootstrap configuration profile.

Therefore, it can be stated that PhyloMissForest provides a robust imputation methodology in terms of the user preference, since it fits not only the users concerned with the accuracy, but also the ones focused on the execution time. The reported experimental results suggest that the proposed framework is a valuable tool to enhance phylogenetic research under missing data constraints. Nevertheless, it opens potential research directions and improvements in the future. For example, the random forest technique is widely used in ML approaches but it can be further improved by integrating parallel strategies that exploit the computing capabilities of modern hardware architectures. Therefore, efficient parallel approaches and implementations on GPU can be developed to turn this model faster [41]. This will result in a boost on the computational performance of our proposal. In order to further improve the accuracy of the imputed phylogenetic distances, especially in scenarios when the NRF increases with the percentage of missing data, a promising approach lies in the hybridization of our current method with other alternative methodologies that operate directly at the alignment level [27]. Another important research topic is the classification of imputation scenarios according to the degree of bias and precision loss introduced by the missing entries, verifying the capabilities of non-bootstrap and bootstrap techniques to conduct accurate imputations under different constraints.

## Conclusions

This work has been focused on defining an ML-based framework to allow the reconstruction of phylogenetic trees from distance matrices with missing data. The devised approach, designated as PhyloMissForest, is built upon random forest based imputation algorithms, which are merged with a number of different search strategies, coupled with phylogenetic techniques and criteria, to efficiently tackle the missing data problem. By adopting the proposed framework, the user is able to customize the imputation process and decide the strategies that best fit the particularities of the input data.

The experimental evaluation revealed that PhyloMissForest is able to fill the gaps identified in the current state-of-the-art ML methods for phylogenetic imputation. The proposed framework attains boosted accuracy in the inferred phylogenetic relationships and improvements in execution time for different real-world evaluation scenarios, in comparison to other previous methods such as matrix factorization and autoencoder. The results herein presented suggest that our approach represents a valuable tool to improve phylogenetic studies in the presence of missing data.

## Methods

The PhyloMissForest framework is built upon a random forest imputation scheme. A random forest can be defined as a group of decision or regression trees. A regression tree is aimed at predicting an output continuous variable based on a set of input features. The first step to build a regression tree is to identify which feature splits the data in two parts with the least error. In order to accomplish this goal, potential split points must be identified. Error measurements are employed to select the points that lead to the most promising splits for each feature. Afterwards, the feature that minimizes error is selected to conduct the dataset splitting, defining a new decision node in the tree. This process is repeated in a recursive way, finding for each subset of the original dataset the best split and creating new decision nodes until the subset under processing cannot be splitted anymore i.e. a leaf node is generated.

**Fig. 7:**
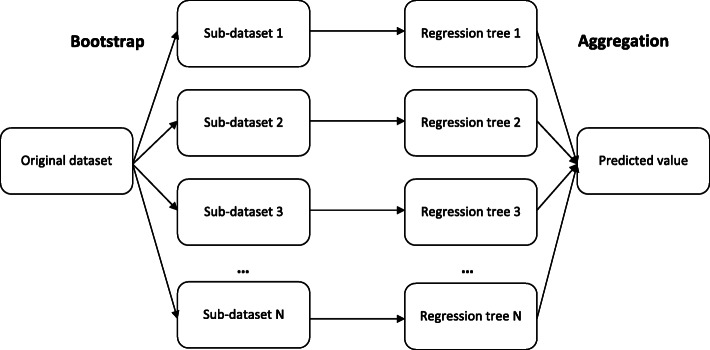
Random forest scheme with bootstrap and aggregation steps

The random forest approach was proposed to deal with the low bias and high variance issues associated to the use of a single regression tree. Under this scheme, different trees are generated with different parts of the input dataset, which are randomly selected. The main idea is to train successive trees with the known parts of the dataset and use the trained random forest to predict the unknown values. When predicting, each tree generates a value for the prediction and the decision outputted by the random forest is given by the average of all predictions, following an aggregation approach. This idea is represented in Fig. 7.

All the search strategies and techniques defined in PhyloMissForest are explained in detail throughout this section.

### PhyloMissForest overview

**Fig. 8:**
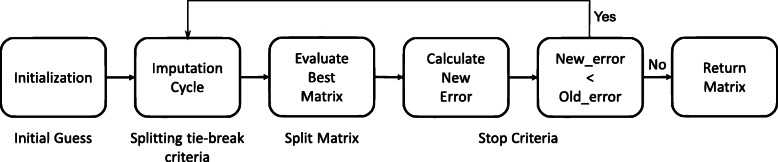
Flowchart of the phases of PhyloMissForest
- ***M***_***old***_ and *M*_*new*_ are the matrices from the previous and current iterations of the imputation cycle, respectively;
- ***old***_***error***_ and *new*_*error*_ are the errors from the previous and current iterations, respectively;
- *X*_*obs*_ and *y*_*obs*_ are the variables used to train the forest, which correspond to the known part of the input data;
- *X*_*miss*_ is the variable used by the trained forest to predict the missing values *y*_*miss*_.

Figure 8 depicts a flowchart of each phase of the imputation methodology devised for PhyloMissForest. The algorithm pseudocode is presented in Algorithm 1. We will focus firstly on describing the main idea of the imputation scheme, which is based on the MissForest approach [37]. Important parameters to understand the behaviour of the referred scheme are as follows:

- *M* is the distance matrix inputted by the user;

In order to know the location of the missing values, the algorithm initializes first a boolean mask with the same dimensions of the inputted distance matrix *M* (line 2 in Algorithm 1). The positions set to the logic value of ‘true’ correspond to the unknown data, while the positions where the logic value is ‘false’ refer to the entries known in the input. Once the creation of the mask has finished, an initial guess for the missing values is calculated. Given a column, the missing values are imputed with an initial guess that corresponds to the mean of the referred column (line 3 in Algorithm 1). This process is repeated for each column in the input. The algorithm then counts the number of missing values per column and stores it in a vector *k*, in order to set the order in which the imputation will occur (line 5 in Algorithm 1). By default, the imputation starts in the column with the least number of missing values. In case of a tie in the number of missing values, the column with the lowest index among the columns that are tied is taken first. The initial values of *old*_*error*_ and *new*_*error*_ are *∞* and 0, respectively (line 6 in Algorithm 1). This is to force the algorithm to run at least two iterations. Therefore, the first iteration is considered as a zero iteration. *M*_*old*_ and *M*_*new*_ are set with a copy of the input matrix *M*.


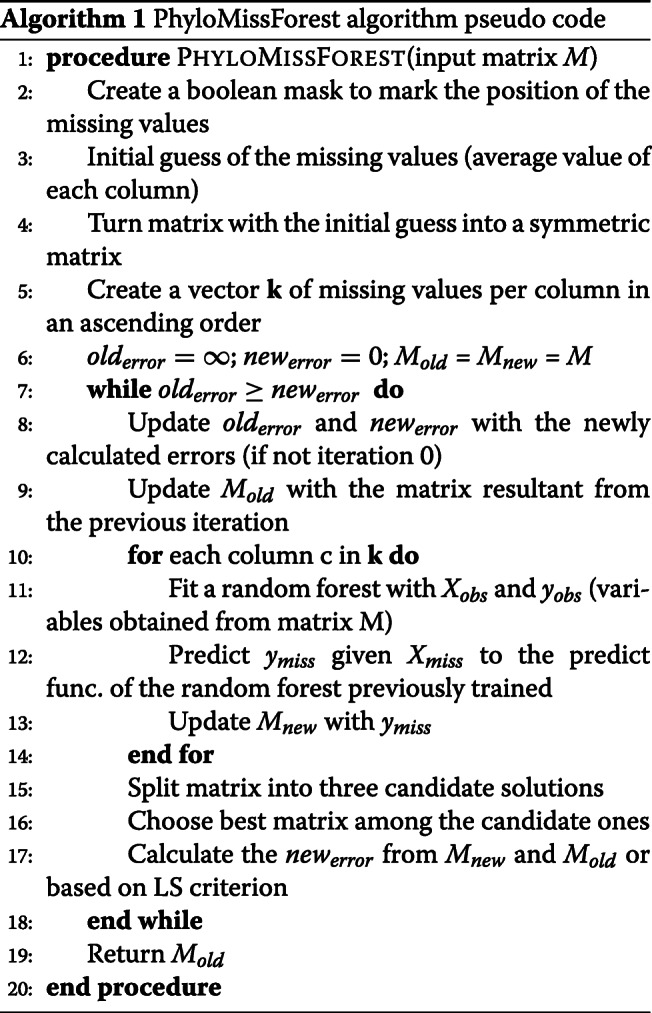


After this initialization process, the imputation loop begins (line 7–18 in Algorithm 1). The first step of the imputation loop lies in the update of the matrix *M*_*old*_ with a copy of the matrix *M*_*new*_. This operation is performed to update the matrix of the previous iteration before the new iteration begins. In the first iteration, this step is ignored because the initialization explained above substitutes this step. After the update of the matrix *M*_*old*_, the algorithm iterates over each column *c* following the order defined in the vector *k* (line 10-14 in Algorithm 1). During the execution of this inner loop, the algorithm employs the following variables: *X*_*obs*_,*X*_*miss*_,*y*_*obs*_ and *y*_*miss*_. A random forest model is fitted with *X*_*obs*_ (values of the other columns in the positions where the currently processed column *c* has observed values) and *y*_*obs*_ (observed values of *c*), given as an input. The trained model uses a predicting function in order to estimate the missing values *y*_*miss*_ from the given *X*_*miss*_. At the end of each iteration of this loop, the predicted values are imputed in the unknown positions of the column *c*, which is the one taken into account in this iteration. This process is repeated for each column in the matrix.

After finishing the imputation of all the columns, the error between *M*_*old*_ and *M*_*new*_ is calculated, e.g. by computing the variable set difference as expressed in Eq. (2) (line 17 in Algorithm 1). The previous *new*_*error*_ is copied to the *old*_*error*_ and the *new*_*error*_ is updated as follows:

2 $$ new_{error} = \frac{\sum_{i,j}^{}(X_{new}[i,j]-X_{old}[i,j])^{2}}{\sum_{i,j}(X_{new}[i,j])^{2}}   $$

If *new*_*error*_ is smaller than *old*_*error*_, the algorithm proceeds with another iteration. Otherwise, the stop criterion is satisfied and the algorithm returns the imputed matrix from the previous iteration, *M*_*old*_, as it gives a smaller error than *M*_*new*_. Additional file 3 provides an example on the use of this methodology.

In order to enhance the imputation capabilities of this scheme for phylogenetic continuous data, the design of the algorithm must be accordingly refined. The following strategies are defined in PhyloMissForest to handle effectively the specific constraints of the phylogenetic imputation problem:

- **Initial guess**: In the initialization step, PhyloMissForest introduces a technique to ensure that, after the initial guess of the missing values, the matrix satisfies the main properties of a phylogenetic distance matrix (line 4 in Algorithm 1);
- **Splitting tie-break criteria**: During the imputation cycle of the PhyloMissForest, the framework defines a method to deal with the ties during the splitting process in the decision trees building. This method is applied in the training phase of each random forest (line 11 in Algorithm 1). Herein our framework not only supports a default configuration based on random selection among the tied features, but also a tie-break criterion based on the Q-matrix selection criterion implemented in the NJ tree-building approaches;
- **Split matrix**: Given that the matrix outputted from the imputation cycle is not symmetric, our framework introduces several strategies to deal with this issue. More specifically, PhyloMissForest explores the possible solutions that can derive from a single matrix by splitting the matrix into three candidate solutions: lower triangular, upper triangular and mean between both (lines 15-16 in Algorithm 1). Moreover, in order to select the best matrix among the candidate ones, PhyloMissForest adopts phylogenetic criteria, such as LS and ME. With the combination of these techniques, our framework not only ensures that all the content available after the imputation is deeply explored, but also that the matrix chosen is the one that best fits a phylogenetic tree;
- **Stop criteria**: The stop criterion of PhyloMissForest also supports the inclusion of phylogenetic LS, ensuring that the algorithm is only concluded if the actual matrix is worse, from a phylogenetic perspective, than the matrix of the previous iteration (line 17 in Algorithm 1).

Throughout the next subsections, a precise description of each strategy included in PhyloMissForest is provided.

### Initial guess

The first strategy included to improve the imputation methodology is to turn the initial guess of the missing values more guided to the tackled problem. Taking into account the properties of a pairwise distance matrix, the matrix outputted from the algorithm needs to be symmetric. In order to help the algorithm to converge to a symmetric matrix, it is helpful if the matrix with the initial guess is also symmetric. Hence, after guessing the initial values for the missing entries, the mean between the distance *i*,*j* and *j*,*i* is calculated and both distances are updated with this average value. With this procedure, we ensure that, when the imputation process begins, the matrix with the initial guess satisfies the symmetric properties of a phylogenetic distance matrix.

### Splitting tie-break criteria

Throughout the imputation cycle, several random forests composed by groups of regression trees are trained. When building a decision tree, in the process of splitting a node into two sub-nodes, a tie between the candidate features can arise. This occurs when two features split the dataset with the same sum of square residuals (SSR), which is the metric used to measure the error given by splitting a dataset based on a particular variable. Our framework supports not only a default configuration based on random choices, but also a decision rule based on the Q-matrix of the NJ algorithm. Q-matrix is an auxiliary matrix defined during the procedure of inferring a phylogenetic tree via NJ, being this matrix used to decide the order of the pairs of OTUs to be agglomerated.

After calculating the initial values *δ* for the missing distances, if the tie-break criterion is configured with the Q-matrix based split decision, the algorithm calculates the Q-matrix corresponding to the distance matrix with the initial guess using Eq. 3. Once obtained the referred matrix, the algorithm defines a list of priorities for each OTU based on the Q-matrix values. For each OTU, the algorithm will search in the Q-matrix which is the other OTU (among the other *N*) that shares the lowest value in the Q-matrix. This will be the first OTU to appear in the list of priorities. The algorithm will repeat the procedure over the remaining OTUs to construct the list of priorities for each OTU involved in the study.

3 $$ Q_{ij}=(N-2) \delta_{ij}-S_{i}-S_{j}, \quad \text {where}\ S_{x}=\sum_{i=1}^{N} \delta_{x i}.   $$

Having the list of priorities defined, if a tie in terms of SSR is observed when the splitting method is in progress, the algorithm will check the list of priorities and choose, from the OTUs that are tied, the one that appears first in the list of priorities of the OTU that is being imputed.

### Split matrix

Similarly to the initial guess process, when the algorithm finishes an entire iteration, after the imputation loop has processed all columns, the resulting matrix may not satisfy the characteristics of a phylogenetic distance matrix. Therefore, we introduce a procedure at the end of the imputation cycle to deal with this issue.

The matrix could simply become symmetric by executing the function developed in the initial guess method. However, since now we have a matrix that is the result of an imputation process, flexibility and accuracy could be lost with that approach. Hence, we developed a new strategy that, from one matrix, examines three possible solutions. As the matrix is not symmetric, the lower and upper triangular parts are different. Therefore, if we assume that each one represents a possible solution and that the mean between both is also a candidate solution, three candidate matrices can be considered instead. We designate this procedure as *split matrix*.

Given these three possible solutions, the problem of how to choose the best distance matrix arises in this context. In order to address it, we merged LS into our proposal. For each pairwise distance matrix, a phylogenetic tree *T* will be constructed. Then, the adaptation of each phylogenetic tree is measured following Eq. (4), where *δ*_*ij*_ represents the distance between the OTUs *i* and *j* in a pairwise distance matrix, while *θ*_*ij*_ refers to the distance between the OTUs *i* and *j* in the phylogenetic tree built from the referred distance matrix. Since LS chooses the matrix where the discrepancy is more subtle, the matrix to be selected is the one that minimizes the value of *S* in Eq. (4).

4 $$ S = \sum_{i,j}^{}(\delta_{ij}-\theta_{ij})^{2}   $$

Merging LS to our proposal allows the imputation method to identify which of the three candidate matrices best fits a phylogenetic tree. Therefore, when all the columns have been imputed, the algorithm performs the following steps:

1. Split the imputed matrix into three possible solutions: lower triangular, upper triangular and mean between both;
2. Build a phylogenetic tree for each possible solution using NJ;
3. Recover the inferred distance matrix from each phylogenetic tree;
4. Calculate the LS values using the matrices of the candidate solutions and the matrices inferred from the phylogenetic trees;
5. Choose the solution that minimizes the value of LS.

Although there is evidence in the state-of-the-art that the ME has issues in certain theoretical scenarios [22], our framework also allows the adoption of this alternative criterion, so that the user is free to choose different decisions throughout the operation. Hence, according to the user settings, our framework allows choosing the best matrix among the three possible solutions by alternatively using the ME criterion. Lastly, the framework also allows the user to choose the best matrix by only comparing each of the three possible solutions with the matrix of the previous iteration. In this case, the algorithm uses the stop criterion expressed in Eq. (2). Once finished the calculation of the three errors, the chosen matrix is given by the one that minimizes the value of Eq. (2). However, if the user applies this alternative configuration, the outputted matrix will be selected without considering information from phylogenetic criteria. Additionally, the user can alternatively discard the process of analysing the three possible solutions. If this step is discarded, the user has the possibility of doing the mean between the lower and upper triangular parts at the end of each column imputation or only when all columns are imputed. However, in this scenario, no phylogenetic criteria will be involved in the selection of the final solution, similarly to the case of using Eq. (2).

### Stop criteria

Finally, our framework allows the user to choose between two different methods to measure the error between the matrix of the previous iteration and the currently imputed matrix. The first consists of applying Eq. (2) as stop criterion, while the second one obtains the error by considering the LS criterion.

The basis of the first criterion is to compare position by position the matrix from the previous iteration and the matrix from the current iteration. By applying this approach, the algorithm evaluates how close the new matrix is to the matrix of the previous iteration by analysing exclusively the values of the matrices. Although the PhyloMissForest framework allows the user to choose this method to obtain the error, it is recommended to select the stop criterion based on LS instead, since it evaluates the phylogenetic trees with the purpose of attaining an improvement in the performance of the imputation algorithm.

### Hyperparameters

When using a ML method, the user usually has to set several hyperparameters that control the algorithm. There are six hyperparameters in the proposed approach:

- **Bootstrap**: This hyperparameter allows the user to enable or disable the bootstrap function. This is a boolean parameter, meaning that it can be set to 0 (non-bootstrap search) or 1 (bootstrap search);
- **Size of the Bootstrap**: This hyperparameter controls the size of the bootstrapped datasets, therefore it is correlated to the previous hyperparameter. The possible values in this case are floating-point numbers between 0 and 1;
- **Max Features**: The aim of this hyperparameter is to define, during the process of building each decision tree, the number of features that are analysed. It supports floating-point values between 0 and 1. The adopted value is multiplied by the size of the dataset to set the percentage of input features to be considered.
- **Min Leaf**: This hyperparameter defines the minimum number of samples that a node has to contain to be considered as a leaf node. It accepts floating-point values between 0 and 1. This value is multiplied by the size of the dataset to accommodate the hyperparameter to the characteristics of the input data.
- **Max Depth**: The aim of this hyperparameter is to control the maximum depth each decision tree can grow. If the user wants to limit the growth of the decision trees, the value to be set to this hyperparameter must be a floating-point number between 0 and 1 (multiplied by the size of the dataset). Otherwise, the value must be -1;
- **Number of trees**: The value of this hyperparameter defines the number of trees of each random forest. It only supports positive integer values and the user is free to choose the number of trees to be included in each forest.

## Supplementary Information

**Additional file 1** Framework Study. Results obtained in the comparative evaluation of the different search strategies integrated in the PhyloMissForest framework.

**Additional file 2** Hyperparameter Study. Results obtained in the parametric studies carried out to configure the hyperparameters of the imputation method.

**Additional file 3** Imputation with Random Forest. An example on the use of random forests for phylogenetic data imputation.

## Data Availability

PhyloMissForest and the real-world datasets supporting the conclusions of this article are available in the GitHub repository, at https://github.com/diogopinheiro13/PhyloMissForest. Requirements: Linux Operating System with compiler GCC version 7.3.0 or above, Python version 3.7.7. The simulated datasets are available at http://www.atgc-montpellier.fr/phyml/datasets.php (40x40) and https://sites.google.com/eng.ucsd.edu/datasets/astral/astral-ii (201x201).

## Abbreviations

HTU: Hypothetical Taxonomic Unit
OTU: Operational Taxonomic Unit
UPGMA: Unweighted Pair Group Method with Arithmetic Means
WPGMA: Weighted Pair Group Method with Arithmetic Means
NJ: Neighbor Joining
FM: Fitch–Margoliash
LS: Least Squares
ML: Machine Learning
RF: Robinson Foulds
NRF: Normalized Robinson Foulds
ME: Minimum Evolution
DOE: Design of Experiments
SSR: Sum of Square Residuals
