# Supplementary material for: PhyloMissForest: a random forest framework to construct phylogenetic trees with missing data
Source: BMC Genomics. 2022 May 18;23:377. doi: 10.1186/s12864-022-08540-6 (PMC9116704; doi:10.1186/s12864-022-08540-6)
Supplement: Supplementary file 1 — Additional file 1 Framework Study. Results obtained in the comparative evaluation of the different search strategies integrated in the PhyloMissForest framework. [file 12864_2022_8540_MOESM1_ESM.pdf]

Evaluation of the framework

In order to tune the hyperparameters of our algorithm, and since our framework has a wide range of possibilities in each decision of the algorithm, a study of the different strategies included in our proposal, with all the parameter set to their default values is performed. In this way, we aim to select the combination of decisions that best fit our datasets. After selecting the combination of decisions, we will proceed with the hyperparameter tunning study.

Taking into account the wide range of possibilities that our framework offers to the user, we divide the study in two main phases:

Component Evaluation Phase: In this phase, we tested the split matrix with the three possible criteria to choose the best solution: Least Squares (LS), Minimum Evolution (ME) or the same expression as the original MissForest. We also test two simpler versions: turning the matrix symmetric after each column imputation and turning it symmetric only at the end of all columns imputation. With this, we test five different ways to choose the best matrix, each one combined with stochastic decisions or Q-matrix based decisions in the process of building each decision tree. In this phase, the stop criteria remains the original one;

Stop Criterion Phase: In this phase, based on the results of the previous one, we evaluate the two stop criteria: original MissForest stop criteria and LS-based stop criteria, that our framework supports in the combinations of decisions that performed better in the first phase. For both the first and second phase, the initial guess of the missing values is performed with our methodology: first, impute the average value of each column and then turn the matrix symmetric.

Framework Study: Component Evaluation Phase

Aiming to evaluate the framework, we run each combination in three datasets with percentages of missing data between 5% and 20%, with increments of 5%. The results of this procedure are presented in Table 1.1. In this Table there are five different versions of the imputation loop decision: **Sym During** where we turn the matrix symmetric during the imputation (after each column imputation); **Sym End** where we turn the matrix symmetric only when all columns are imputed; **Split-O** where we analyse the three possible matrices with the same expression as the stop criterion; **Split-LS** where we analyse the three possible solutions by coupling LS; **Split-ME** where we analyse all the three possible solutions by coupling ME criteria. For each of the five enumerated versions, the two possible tie-break criteria are tested: random decisions and Q-matrix decisions, designated as **R** and **Q**, respectively.

For each dataset and percentage of missing data, the version of the algorithm that obtained the lowest value of Normalized Robinson Foulds (NRF) is claimed the winner, and the result in Table 1.1, is in bold. In order to better understand the behaviour of each combination, for each percentage of missing data, 5 different matrices are tested. For example, for the dataset 9x9 with 5% of missing data, the value of NRF provided in the Table 1.1 is the average of the NRF obtained in all of the 5 matrices. We also show the total number of wins of each version and also the referred value in percentage. In order to have a general perspective of each combination of decisions, we also present in Table 1.1 the sum of all

the NRF average values.

Table 1.1: NRF values for all the versions of the algorithm in the first phase of the framework study.

Data	% MD	Sym During		Sym End		Split-O		Split-LS		Split-ME	
		**R**	**Q**	**R**	**Q**	**R**	**Q**	**R**	**Q**	**R**	**Q**
9x9	5%	6.7%	6.7%	7.5%	8.3%	6.7%	8.3%	**5.0%**	8.3%	11.7%	8.3%
	10%	12.5%	13.3%	14.2%	12.5%	12.5%	13.3%	**11.7%**	16.7%	20.0%	13.3%
	15%	12.5%	**6.7%**	10.8%	10.0%	9.2%	13.3%	10.8%	10.0%	12.5%	8.3%
	20%	20.0%	15.0%	21.0%	13.3%	14.2%	13.3%	14.2%	**11.7%**	19.2%	16.7%
37x37	5%	4.6%	3.8%	4.0%	3.2%	4.9%	**2.4%**	2.8%	2.6%	7.6%	4.1%
	10%	4.3%	**2.1%**	4.3%	4.7%	7.2%	5.9%	3.8%	3.2%	5.0%	4.7%
	15%	7.8%	**5.6%**	7.4%	9.7%	5.9%	6.2%	6.2%	7.9%	8.8%	6.5%
	20%	10.3%	15.3%	10.3%	15.9%	13.5%	13.2%	8.2%	**7.4%**	13.8%	14.7%
55x55	5%	4.5%	4.6%	3.2%	5.0%	4.0%	3.8%	**2.3%**	4.8%	4.2%	5.2%
	10%	13.4%	16.2%	15.5%	19.0%	14.5%	18.3%	**11.2%**	15.4%	16.2%	17.3%
	15%	14.5%	15.4%	13.6%	13.7%	13.0%	11.7%	11.5%	**9.4%**	15.0%	13.8%
	20%	17.3%	16.5%	18.6%	18.7%	17.4%	17.4%	**15.0%**	18.3%	20.4%	20.4%
SUM NRF		128.3%	121.1%	130.2%	134.0%	122.9%	127.2%	**102.7%**	115.7%	154.5%	133.4%
WINS		0	3	0	0	0	1	5	3	0	0
%WINS		0%	25%	0%	0%	0%	8%	**42%**	25%	0%	0%

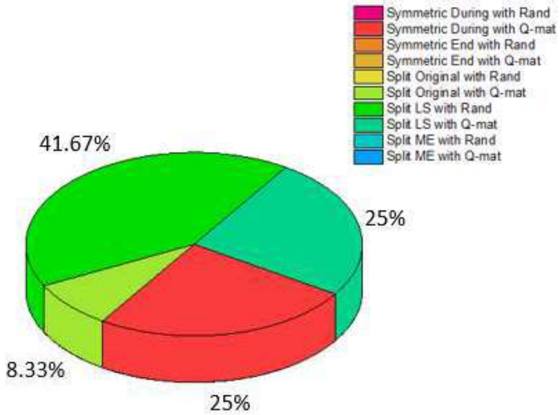

Figure 1.1: Percentage of wins for each combination of the first phase of the framework study.

From the analysis of Table 1.1 and Figure 1.1, we can draw some conclusions:

- The version that obtains better results is the split version, coupled with LS and using stochastic decisions;
- In a more general perspective, the split version with LS obtains 67% of wins, more precisely, 42% from the version with stochastic decisions and 25% from the version based on Q-matrix decisions;
- The version that turns the matrix symmetric at the end of each column imputation, for the case in which the ties are solved with Q-matrix decisions, achieves 25% of wins;
- Both the version based on the ME criteria and the version that turns the matrix symmetric at the end of the imputation of all columns do not get any win;
- The version that analyses the split of the three matrices using the same method as the stop criteria only gets 8% of wins.

Given the reasons described above, the next phase of the study of the framework, will focus on the versions that analyse the split based on LS, both the random and Q-matrix decisions. The version that

turns the matrix symmetric during the imputation loop with Q-matrix decisions also passed to the next phase. In terms of the split that analyses the three solutions with the same expression as the stop criteria and solves the ties with Q-matrix decisions, it is considered as an outlier since this version only gets one win. For all the other versions, they do not get any win, therefore, their results are not considered enough to pass them to the second phase of the evaluation of the framework.

Framework study: Stop Criterion Phase

During the second phase of testing the performance of the framework, for the three versions that passed in the previous phase, we test two different stop criterion, **Orig**, which is the MissForest original stop criterion and **LS**, which is a stop criterion based on LS. Due to the fact that the split version coupled with LS, both with stochastic and Q-matrix based decisions wins the majority of the runs in the first study, in this phase we also test a hybrid version called **Split-LS-Hybrid**, where there is a mix between stochastic and Q-matrix decisions. For this last version, the two stop criteria explained above are also tested.

In order to evaluate each version of the second phase of the study of the framework, we run each version in three datasets with percentages of missing data between 5% and 30%, with increments of 5%. Aiming to better understand the way each combination behaves, we use the same procedure used when testing the first phase of the study, meaning that, for each percentage of missing data, 5 matrices are tested, and the NRF for a specific percentage is the mean between the NRF of the 5 matrices**.** The results of the refereed process are provided in Table 1.2. Aiming to have an overall overview of each combination of decisions, we also present in Table 1.2 the sum of all the NRF average values. Moreover, to be possible to compare our framework solutions with the baseline MissForest, the latter is presented in the last column of the Table 1.2.

Table 1.2: NRF values for all the versions of the algorithm in the second phase of the framework study.

Data	% MD	Split-LS-Rand		Split-LS-Q		Split-LS-Hybrid		Sym During Q		Baseline
		Orig	LS	Orig	LS	Orig	LS	Orig	LS	
9x9	5%	**2.9%**	4.6%	3.8%	5.0%	4.2%	5.0%	4.6%	4.2%	5.0%
	10%	7.1%	7.5%	7.9%	7.9%	8.7%	**6.7%**	9.6%	7.5%	11.7%
	15%	9.6%	**7.5%**	10.0%	10.4%	9.6%	8.3%	9.6%	9.6%	12.1%
	20%	13.8%	12.5%	10.4%	**9.2%**	10.8%	9.6%	12.5%	12.5%	13.8%
	25%	13.8%	**12.5%**	15.4%	14.2%	12.9%	16.3%	19.2%	17.1%	19.2%
	30%	17.1%	**12.9%**	16.3%	15.0%	15.4%	13.8%	17.1%	14.6%	17.9%
37x37	5%	1.7%	**1.2%**	1.3%	1.3%	1.5%	1.3%	2.5%	2.1%	2.0%
	10%	3.8%	**2.8%**	5.4%	4.6%	5.5%	3.5%	3.9%	3.2%	4.6%
	15%	**4.6%**	**4.6%**	5.7%	5.1%	5.8%	5.4%	10.1%	8.3%	7.0%
	20%	**6.5%**	6.8%	8.7%	9.0%	9.6%	6.6%	13.2%	11.0%	9.4%
	25%	9.6%	**9.3%**	12.3%	11.4%	10.6%	9.6%	15.4%	14.6%	14.0%
	30%	14.4%	**13.2%**	16.2%	15.7%	15.9%	15.8%	20.4%	17.9%	16.2%
55x55	5%	4.6%	3.4%	4.1%	3.1%	4.6%	**3.0%**	5.6%	3.9%	6.3%
	10%	7.2%	5.9%	8.0%	7.0%	6.9%	**5.7%**	10.1%	9.4%	8.7%
	15%	12.6%	11.0%	14.2%	12.6%	13.5%	**10.1%**	17.9%	14.7%	14.7%
	20%	16.9%	**14.3%**	16.7%	15.1%	17.6%	15.0%	19.1%	14.5%	16.9%
	25%	22.3%	20.5%	22.1%	20.7%	23.8%	**19.7%**	25.2%	22.5%	22.0%
	30%	24.6%	**22.3%**	26.7%	25.0%	25.7%	23.5%	29.0%	26.3%	25.0%
SUM NRF		192.9%	**172.8%**	205.3%	192.3%	202.7%	178.9%	245.0%	213.9%	226.3%
WINS		2.5	9.5	0	1	0	5	0	0	0
%WINS		14%	**53%**	0%	6%	0%	28%	0%	0%	0%

Note that in the dataset with 37 Operational Taxonomic Units (OTU) with 15% of missing data, the

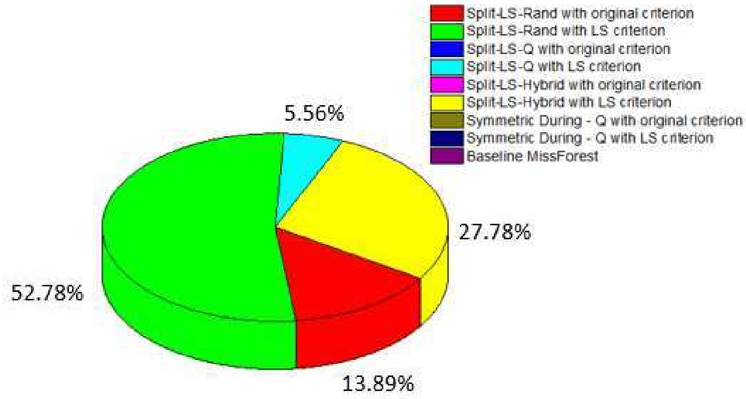

Figure 1.2: Percentage of wins for each combination of the second phase of the framework study.

version that analyses the split with LS and the ties with stochastic decisions both with the original criteria and with the LS criteria, get the same value of NRF. Therefore, both win in this particular case. In order to be possible to draw the diagram of Figure 1.2, the sum of the victories has to be 100%. Hence, we divide the win between both versions, each one getting 0.5 in the cumulative number of wins. Taking into account the described procedure, the split-ls-rand version with the original stop criterion takes 2.5 wins, while the split-ls-rand version with LS stop criterion gets 9.5 wins.

From the analysis of Table 1.2 and Figure 1.2 we can draw some conclusions:

- The inclusion of the stop criterion based on LS, increases the accuracy of each algorithm when comparing to the ones that use the original stop criterion. This is proved not only in the number of wins, but also in the sum of the NRF.
- The versions that analyse the split between the three matrices outperform the ones that turn the matrix symmetric during the imputation cycle.
- All the versions devised in the second phase of the study of the framework outperform the baseline MissForest.
- The versions that use stochastic decisions to solve the ties have better results than the version that use Q-matrix decisions, thus denoting the relevance of introducing stochastic techniques to enhance the exploration of candidate solutions.

In order to study the behaviour of each parameter of the algorithm, we need to select one combination of decisions for our framework. Combining the results of both phases of the study of the framework, we can proceed with the study of the parameters in the version split-ls-rand with the LS stop criteria. Recall that in this version, the decisions the algorithm are: the analysis of the three matrices and the selection of the best matrix using LS; solving the ties during decision trees building by using random decisions; and using LS as stop criterion. This version is chosen because it wins not only in terms of the sum of the NRF, but also in the number of wins (over 50%). Nevertheless, all the other versions of our framework

are competitive, since all of them outperform the baseline MissForest in terms of the sum of the mean NRF values present in Table 1.2.
