# Supplementary material for: PhyloMissForest: a random forest framework to construct phylogenetic trees with missing data
Source: BMC Genomics. 2022 May 18;23:377. doi: 10.1186/s12864-022-08540-6 (PMC9116704; doi:10.1186/s12864-022-08540-6)
Supplement: Supplementary file 2 — Additional file 2 Hyperparameter Study. Results obtained in the parametric studies carried out to configure the hyperparameters of the imputation method. [file 12864_2022_8540_MOESM2_ESM.pdf]

Hyperparameter Tunning

In every Machine Learning (ML) method, testing and defining hyperparameters, which is also called as tunning hyperparameters, is one of the most challenging tasks to perform. The most commonly used technique is grid search, in which the user defines a set of values for each parameter and then an exhaustive test of stochastic combinations is tested. This approach can incur in a large number of runs, turning it highly inefficient. Aiming to turn this task more methodological, a Design of Experiments (DOE) is applied.

PhyloMissForest supports six parameters. In terms of the size of the bootstrap parameter, it only has influence if the bootstrap parameter is set to true. The other four parameters are independent of the bootstrap. Hence, we divide the study of the parameters in two parts: when bootstrap is set to false and when bootstrap is set to true. Because of this division, in the first case, we only have four parameters apart from the bootstrap one, whereas, in the second case, we have five parameters apart from the bootstrap, since we have the size of the bootstrap parameter when bootstrap is set to true.

In order to be possible to build a DOE, we use a software powered by *Tibco* called *Statistica*, where the range of values for each parameter has to be defined, more precisely, the software asks the user to insert the lower and upper boundary values of each parameter. Therefore, before inputting the values in the software, we perform a small test for each parameter, where we fix all the others and test a range of values for this parameter. When the range for each parameter is already tested, we start with a DOE, called factorial design of experiments, in order to understand which parameters are more statistically significant in terms of our aim, that is to minimize the Normalized Robinson Foulds (NRF). After that, we will select the three parameters that have the strongest statistical meaning and for the excluded parameters, we fix a value based on the output reported by the software. With the three parameters that remain to be defined, we begin another DOE called Box–Behnken design. The difference between the DOE's is that in the second design, we not only test the boundaries of the range, but also the centre point is included in the combinations tested. This way, we are increasing the depth of our analysis for the parameters that are considered to influence more our goal. Moreover, by testing three values for each parameter, we are performing a quadratic study, while in the first design with just two values, the study is linear.

In conclusion, we divide our analysis in two cases: bootstrap = False, which we call **non bootstrap case** and bootstrap = True, which we call **bootstrap case**. For each of the two versions presented above, a study composed of three phases is performed:

1. Study parameter by parameter, aiming to understand reasonable ranges for each one;
2. Factorial DOE to filter which are the three parameters that have the strongest statistical meaning;
3. Box-Behnken design to define the values of the parameters that remain to define from the previous design;

Using this methodology, in the first phase, we filter the range that makes sense to test for each parameter. In the second phase, we perform a factorial DOE to filter which parameters should we

investigate deeper, and for the others (the ones that are not among this group), we fix values based on the results. Finally, in the third phase, we perform a more complete design in order to determine the values for the three parameters that pass to this phase.

Inspecting the list of possible values for each parameter, we conclude that there are parameters that are defined based on the size of the dataset (size of the bootstrap and max features), while there are other parameters that are not defined by connecting their values with the dataset in usage (min leaf, max depth, number of tree and bootstrap). Since during the experimental evaluation of our algorithm we use three datasets with different sizes, and we also want to fix a combination of parameters that fits all datasets, for the parameters that are not yet defined in function of the size of the dataset, min leaf and max depth, we define them taking it into account. With this methodology, only the number of trees and the bootstrap are not defined in function of the size of the dataset in usage. When we say that we are defining a parameter in function of the size of the dataset, it means that the value of the parameter is going to be a floating point number, between 0 and 1, that is multiplied by the size of the dataset to define the real value of this parameter. The expression used to calculate the presented process is presented in (1.1), where the dim represents the dimension of the dataset and the $perc$ represents the percentage defined for the parameter.

$$Parameter = int(dim \times perc) \tag{1.1}$$

For example, if we are using the 37x37 dataset and we define that the min leaf parameter is 0.3, by applying the expression (1.1), the value for min leaf that is inputted in the algorithm is 37x0.3 = 11.1. However, the min leaf parameter only allows integer values greater than 0. To overcome this problem, we introduce the integer value of the multiplication 37x0.3 that is 11.

Non Bootstrap Configuration

The first step of this study is to understand the range of values that are reasonable to give to the software in the next phase. To do this, we vary parameter by parameter while keeping all the others with their default values. The range of values tested for each parameter is available in Table 1.1.

Table 1.1: Range of parameters tested in the first phase of the study of non bootstrap case

**Parameter**	**Range Tested**
Bootstrap	0 = non bootstrap
Number of Trees	10, 20, 30, 40, 50, 60, 70, 80, 90, 100
Max Features	0.25, 0.5, 0.75, 1
Max Depth	0.1, 0.25, 0.5, 0.75, 1
Min Leaf	0.01, 0.05, 0.1, 0.25, 0.5, 0.75, 1

As it was described, the idea is to go parameter by parameter and try to understand the range of values that is reasonable to test. For example, when testing the max features parameter, aiming to understand its own behaviour, all the others are fixed to the values their default values, while max features is tested between the values defined in Table 1.1. Note that the bootstrap parameter is the only parameter that is not going to vary, since we divide the study between non bootstrap and bootstrap.

Furthermore, the size of the bootstrap is also not present, as in the non bootstrap case the value of this particular parameter is irrelevant. We tested each parameter in the three datasets with percentages varying between 5% and 20% of missing data, with increments of 5%. Since this version is performed only to have an idea of the suitable parameter ranges, we only test one matrix per percentage. In conclusion, the values of NRF presented in Figure 1.1 are the average of the NRF of the twelve matrices herein tested (3 files x 4 percentages x 1 matrix per percentage).

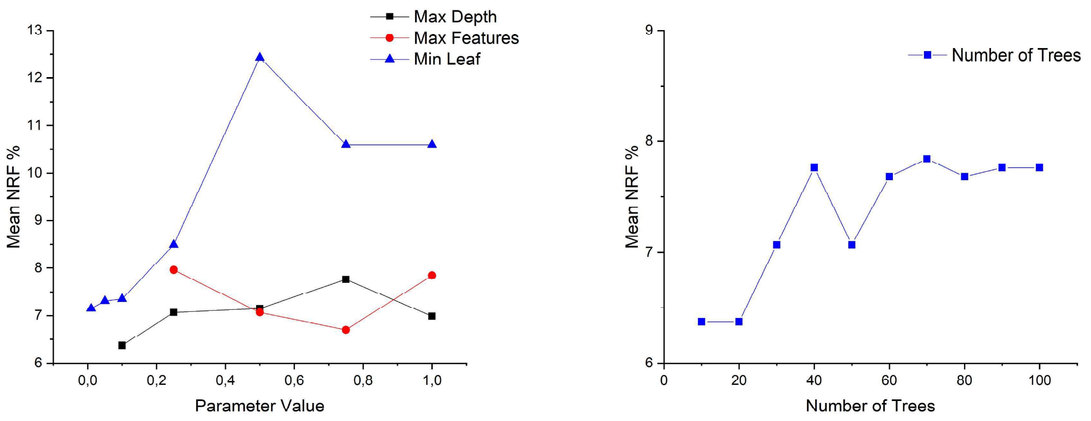

Figure 1.1: Results of the first phase of the non bootstrap case.

In order to define the ranges for each parameter, we analyse the plots present in Figure 1.1. Starting from the plot on the left, we have the three parameters that are defined as a percentage of the size of the dataset. As it is pictured, the parameter min leaf is the only one that has a clear behaviour, since, with the increase of the referred parameter, the value of the NRF increases. This behaviour is reported by the shape of the blue line, where we can see that the values of NRF are worse when the value of the min leaf parameter increases. Hereupon, we define the range of the min leaf between 0.01 and 0.25. Regarding the other two parameters, max depth and max features, there is not a clear behaviour for both of them. As our aim with this first phase is to cut the range tested, only if possible, and here we have a situation where it is not possible to do this, we define that: the range of the max features is between 0.25 and 1 and the range of the max depth is between 0.1 and 1. In terms of the plot on the right of Figure 1.1, with the exception of the random forest with 50 trees, it appears that with an increase of the number of trees in each random forest, there is an increase of the NRF. Due to that fact, we define the range of the number of trees between 10 and 50.

Finished the process of defining the ranges for each parameter, the second phase of the hyperparameter study begins. In this phase, the first DOE is going to be built. Since we have 4 parameters in this study, we build a design of 16 combinations(2^{4-0}). Recall that this design only tests the boundary values, so in order to give information of the central values, 4 central points are tested. A central point is a point where all the parameters are defined with the central value of their range. We insert in the software four central points, as our algorithm introduces some degree of randomness and because of that, running four times the same combinations of parameters does not give the same results, yet they

should be close.

We run each combination of parameters in the three datasets with percentages of missing data between 5% and 20%, with increments of 5%. For each percentage of missing data, 5 matrices are generated. Therefore, we are running each combination in 3 files, with 4 different percentages of missing data and with 5 matrices per percentage, which turns into a total of 60 matrices. Since we defined each parameter in function of the size of the dataset, we can sum the NRF of all the matrices and input this value in the software, as the NRF for each combination. The 16 combinations plus the 4 centre points and each NRF value are presented in Table 1.3.

When inputting in the software the NRF value for each combination, a statistical test called *ANOVA*, with an interval of confidence of 95%, is performed. *ANOVA* is a statistical method that analyses the effect of various factors on some response. A Pareto chart that shows the standardized effect estimate not only for each parameter, but also for the interaction between each pair of parameters, is presented in Figure 1.2. The standardized effect estimate is a statistical measure of the influence of each parameter or interaction between each pair of parameters, in the goal variable, in our case NRF. A more detailed report of the study can be found in Table 1.4, where the parameters or interactions between two parameters that obtained a p-value lower than 0.05 (interval of confidence of 95%) are in bold.

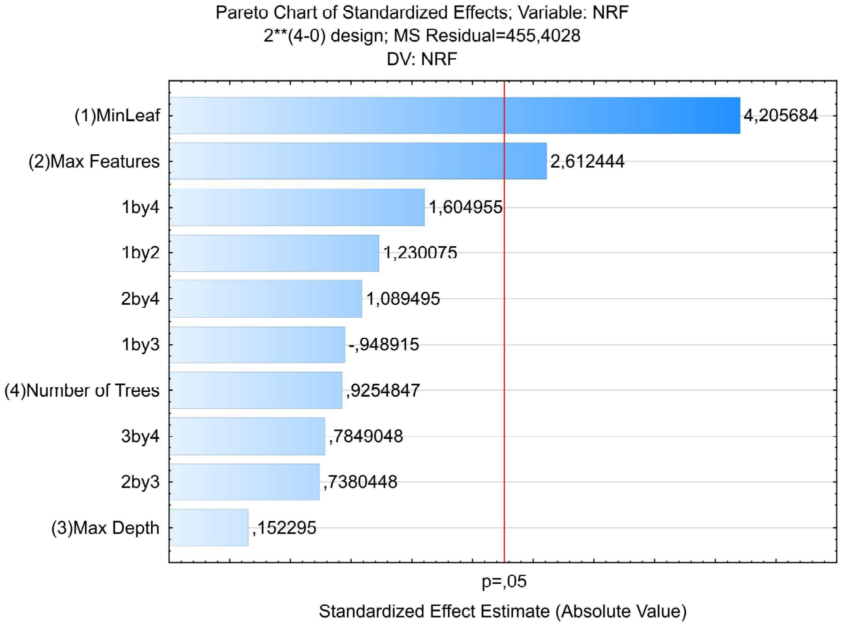

Figure 1.2: Pareto chart of the standerdized effect estimate of each parameter and interaction between parameters for the non bootstrap case.

Recurring to the Pareto chart in Figure 1.2, we can observe that both min leaf and max features have a strong effect on the NRF value, since both are in the right side of the p-value 0.05 line. Therefore, both remain to study in a deeper perspective in the last phase of the non bootstrap study. The box-behnken design of the third phase requires a minimum of three parameters to be built. Therefore, we need to select a third parameter between the number of trees and the max depth. Among those two, we select the number of trees, not only because the effect of this parameter is higher than the max depth, but also because the interactions between the number of trees parameter with the other parameters have more

influence in the NRF than the interactions promoted by the max depth parameter.

Finished the process of choosing the three parameters to build the last design, the values of the parameters that are not going to pass to the next phase, in our case max depth, need to be fixed. As it is depicted in Figure 1.3, by varying this parameter between 0.1 and 1, the difference between the results is not clear. Additionally, the standard deviation (boundaries bars in the plot) is also huge, which turns this parameter into a parameter with less statistical meaning than the others. With this information and knowing that by setting this parameter to 0.1 we are limiting the growth of the decision trees, while by setting it to 1 we are not imposing any restriction on the way the trees are growing, it was decided to fix max depth to 1.

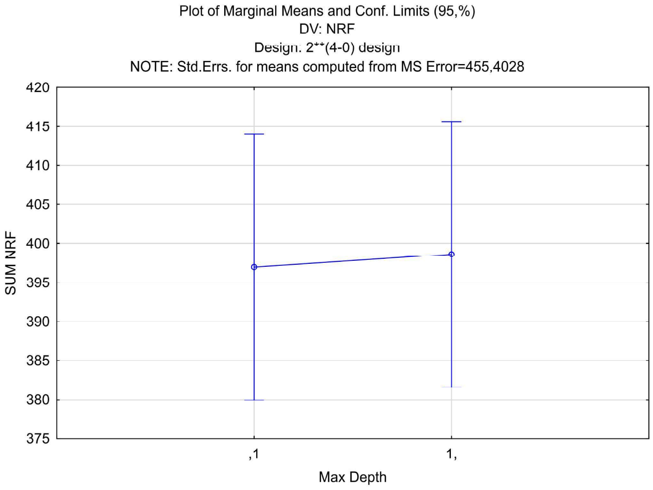

Figure 1.3: Effect of the max depth parameter in the NRF.

Finished the first DOE, with the three parameters that remain undefined, a new design called box-behnken is built. With this design we aim to include, not only the boundaries of the range, but also the central points in the combinations. All the combinations of parameters are tested in the same datasets of the first design. The referred combinations accompanied with the resultant sum of NRF of all matrices are presented in Table 1.5.

As it was explained before, during this second design, the objective is to define the value for each one of the parameters. In order to accomplish this objective, the evolution of the NRF for the three values tested in each parameter is presented in Figure 1.4.

Starting from the upper left graphic in Figure 1.4, which depicts the effect of the max features in the NRF, this parameter confirms its effect in the Pareto chart of the first design. We can see that with the decrease of the value of the max features, there is an increase in the performance, since the NRF decreases. Therefore, the value fixed for max features is 0.25. In terms of the upper right graphic in Figure 1.4, which depicts the effect of min leaf in the NRF, the behaviour of this parameter is clear and also confirms the results of the first phase of the study, where we saw that the NRF increases with the increase of the min leaf. Because of this behaviour, this was in the first DOE, the parameter with strongest standardized effect, since by varying it, we have significant differences in the NRF values. From the results, the final value of this parameter is 0.01. Finally, the number of trees parameter, which

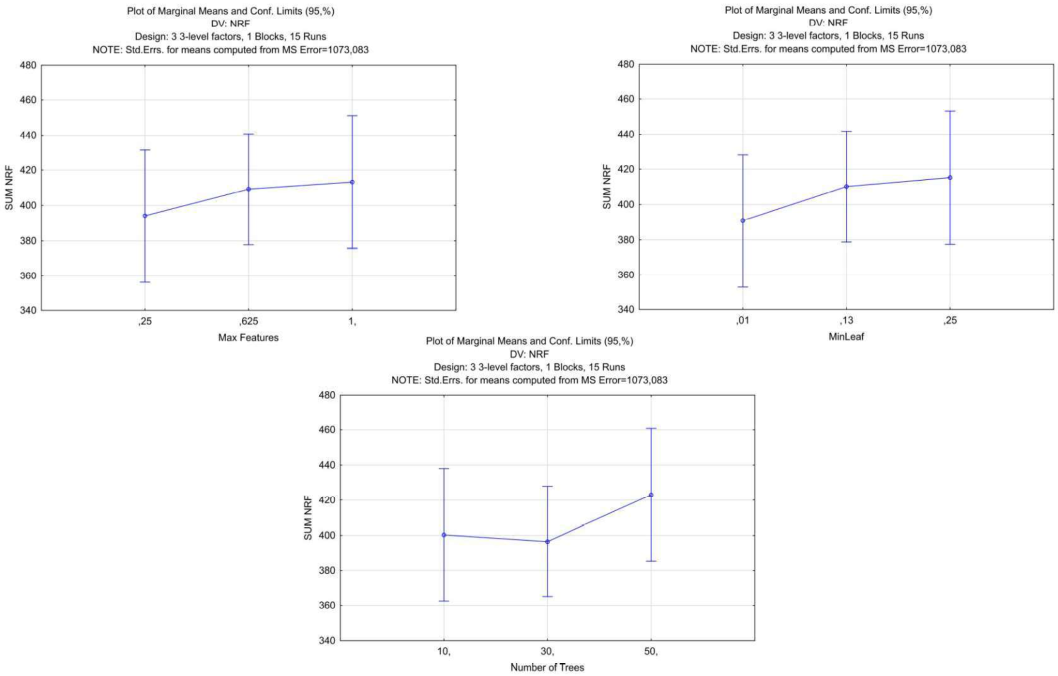

Figure 1.4: Results of the second design for the non bootstrap case.

is presented in the bottom graphic of Figure 1.4, confirmed by the shape of the line in the plot to be, among the three parameters in study during this final stage, the parameter where the results are less clear. Despite this, we can observe that we have less standard deviation and better NRF value with 30 trees. Hence, this value is the final one for this parameter.

Throughout the three phases of the study of the non bootstrap case, decisions about the ranges and values of the parameters were taken. The main objective of this study was to select a combination of parameters optimal for our datasets. In conclusion, the final values for the non bootstrap configuration are: **bootstrap = False; number of trees = 30; max depth = 1; min leaf = 0.01; max features = 0.25.**

Bootstrap Configuration

Similarly to the non bootstrap case, we study the same three phases for the bootstrap case. Starting in the first phase, we are going to test parameter by parameter in order to have an idea of the reasonable ranges for each one. Note that in this case, we join the size of the bootstrap parameter to the list of parameters in study. The range of values tested for each parameter is present in Table 1.2.

Table 1.2: Range of parameters tested in the first phase of the study of bootstrap case.

Parameter	Range Tested
Bootstrap	1 = bootstrap enabled
Size of the Bootstrap	0.2, 0.3, 0.4, 0.5, 0.6, 0.7, 0.8, 0.9, 1
Number of Trees	10, 20, 30, 40, 50, 60, 70, 80, 90, 100
Max Features	0.25, 0.5, 0.75, 1
Max Depth	0.1, 0.25, 0.5, 0.75, 1
Min Leaf	0.01, 0.05, 0.1, 0.25, 0.5, 0.75, 1

During the first phase of the study of the bootstrap case, we use the same procedure employed in

the non bootstrap case. The results of this first phase of study are illustrated in Figure 1.5.

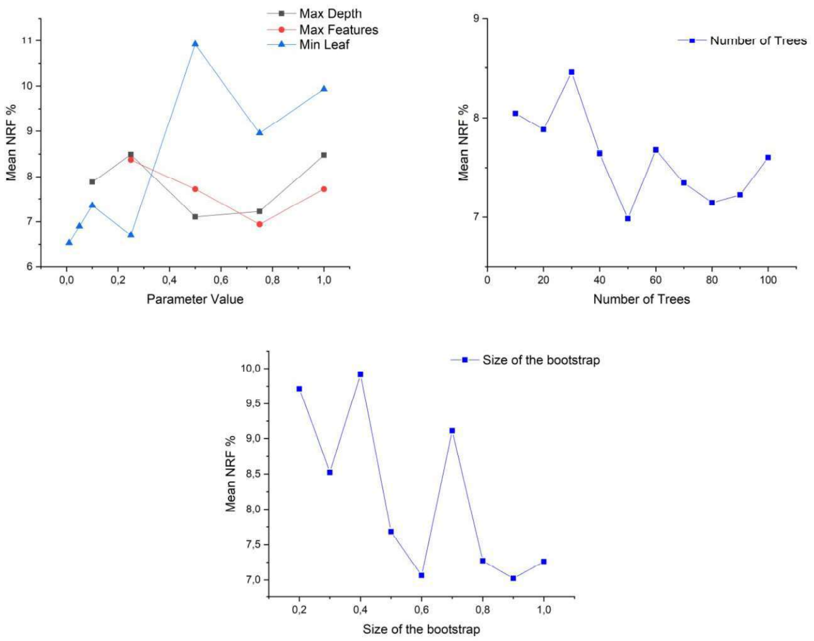

Figure 1.5: Results of the first phase of the bootstrap case.

Starting from the upper left plot in Figure 1.5, we can see a similar behaviour of the parameters included in it. For max depth and max features, as the shape of their curves is not clear, we can not cut the range tested for each one. Therefore, for max features, the range that is going to the next phase is between 0.25 and 1, whereas for max depth parameter, the range is between 0.1 and 1. In terms of the upper right graphic in Figure 1.5, we can see a different behaviour when comparing with the non bootstrap case. Here, we can conclude that with an increase in the number of regression trees of each random forest, the NRF decreases. Hence, we set the number of trees between 50 and 100. In order to analyse the size of the bootstrap parameter, we should inspect the bottom plot of Figure 1.5, where it is depicted the influence of this parameter in the NRF value. Analysing the referred graphic, we can observe that with the increase of the size of the bootstrapped datasets, we have an improvement in the performance of our algorithm, since the mean value of the NRF decreases. From these results, it can be defined the range of this parameter between 0.6 and 1.

Finished the process of defining the ranges for each parameter, we move to the second phase of the study, where the first DOE is going to be built. In this case we have 5 parameters in study, so a DOE of 2^{5-0} would give 32 combinations. Since this is a large number, and in this phase we only want to filter which parameters should we study in a more deeper perspective, we only test 16 combinations, which gives a DOE of (2^{5-1}). For the same reasons presented in the non bootstrap case, we inputted into the software 4 central points. We run each combination generated by the software in the same datasets as the non bootstrap case. As we defined each parameter in function of the size of the dataset, we can sum the NRF of all the matrices and input this value as the NRF for each combination. The 16 combinations plus the 4 centre points and each NRF value are presented in Table 1.6.

Inputting into the software the results of the sum of NRF value for each combination, the statistical study applied in the non bootstrap case is again applied to the bootstrap one. The Pareto chart that shows the standardized effect estimate, not only for each parameter, but also for the interaction between each pair of parameters, is given by Figure 1.6. A more detailed report of the study is available in Table 1.7, where the parameters or interaction between two parameters that obtained a p-value lower than 0.05 (interval of confidence of 95%) are in bold.

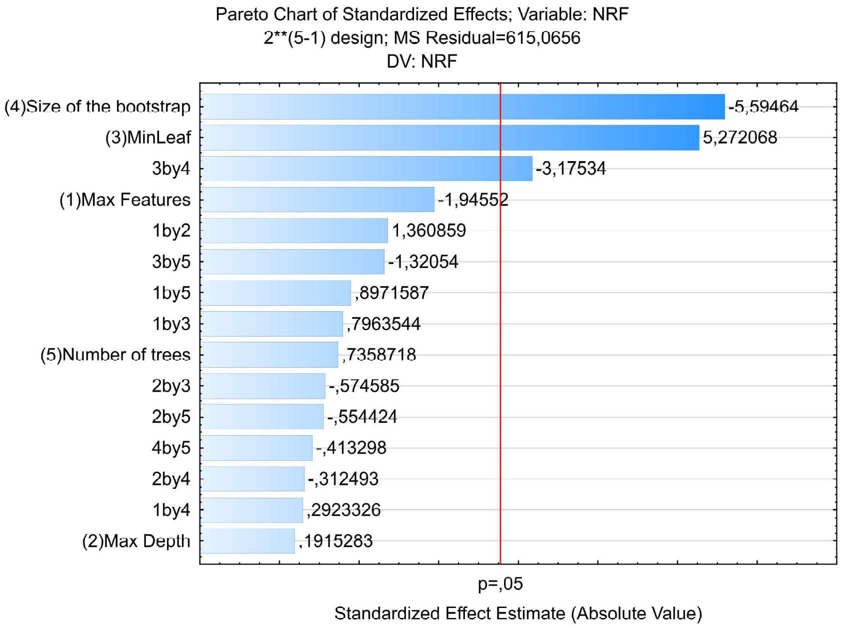

Figure 1.6: Pareto chart of the standerdized effect estimate of each parameter and interaction between parameters for the bootstrap case.

From the Pareto chart presented in Figure 1.6, it can be observed that both size of the bootstrap and min leaf standardize effects reach the right side of the 0.05 p-value line. Moreover, the interaction between these two also achieved a p-value lower than 0.05. Due to that fact, these two parameters are kept with undefined value for the final stage of the study. Immediately after the parameters or connections that reached a p-value less than 0.05, it can be found the max features parameter. The interactions between this parameter and the others are also in the top of the list of the connections, in terms of standardized effect. Combining these two facts, max features is the third parameter that moves to the box-behnken design.

For the parameters that will not move to the next phase, in this case max depth and the number of trees, their values needs to be fixed. Aiming to accomplish this task, the effect of these two parameters in the NRF is shown in Figure 1.7. Starting with max depth, which is represented in the left side of Figure 1.7, we can identify a similar behaviour when comparing to the non bootstrap case. Hence, for the same reasons previously discussed in the non bootstrap case, it is established the value of this parameter to 1. In terms of the number of trees parameter, depicted in the right plot of Figure 1.7, by varying this parameter between 50 and 100, we can observe better results when using 50 regression trees in each random forest. Therefore, in the next phase, the number of trees is fixed to 50.

Finally, a new box-behnken design, is built with the aim of defining the values of the three parameters

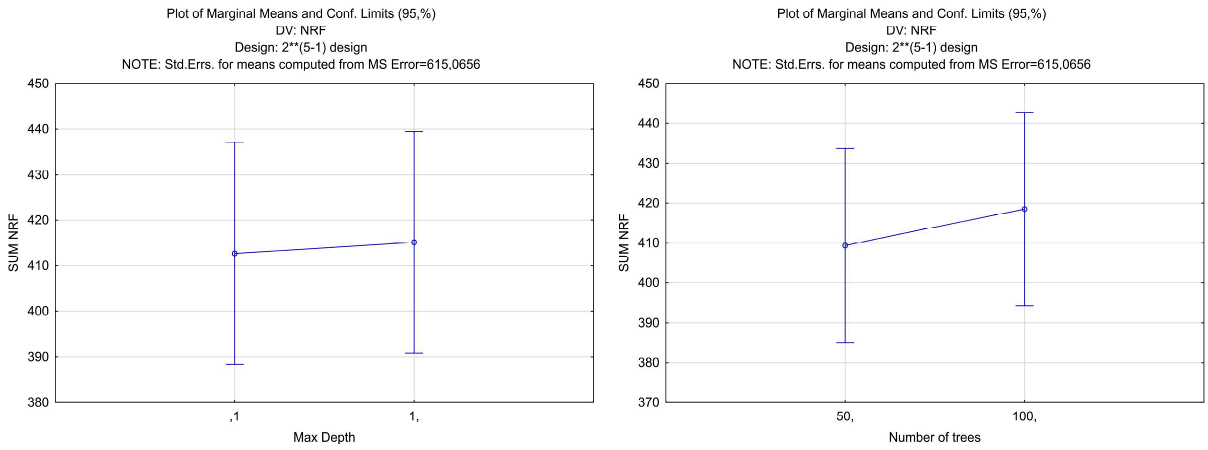

Figure 1.7: Results of the first phase of the bootstrap case.

that revealed to be the ones with most influence in the NRF. With this design we can verify, not only the boundaries of the range, but also the central points in the combinations. We test all the combinations in the same datasets of the first DOE. The referred combinations accompanied with the resultant sum of NRF of all matrices is presented in Table 1.8. As it was explained before, during this second design the objective is to define the value for each of the three parameters, and in order to accomplish this objective, the evolution of the NRF for the three values tested in each parameter is presented in Figure 1.8.

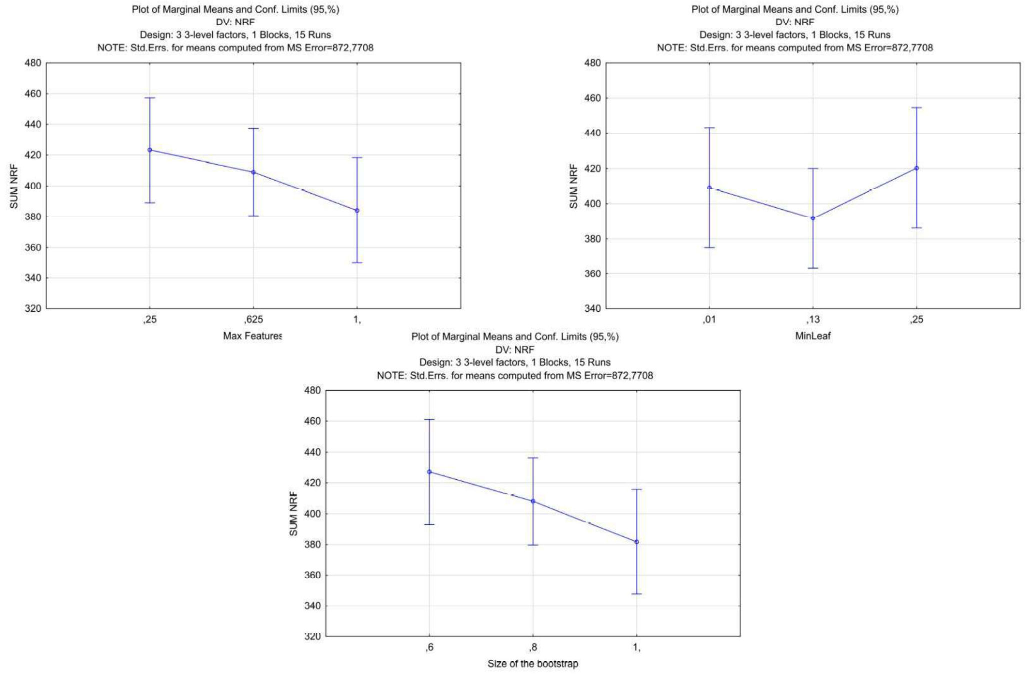

Figure 1.8: Results of the second design for the bootstrap case.

Starting from the upper left graphic, we can see that, when max features parameter increases, the performance of our algorithm also improves. More precisely, we get the minimum value of cumulative NRF, when max features is set to 1. Due to that fact, we set max features to 1. Moving to the upper right

graphic in Figure 1.8, the behaviour of the min leaf parameter in the three points tested is represented. As we can see, the min leaf parameter obtains less cumulative NRF when is set to 0.13 and therefore, the value of this parameter is fixed to 0.13. Finally, in the bottom part of Figure 1.8, it can be observed that, if we increase the size of the bootstrapped datasets, we attain a decrease in the NRF value, and therefore, an improvement on the performance. The results of this parameter confirmed the results of the first phase shown in Figure 1.5. Hence, we fix the value of the size of the bootstrap parameter to 1.

Across the three phases of the study of the bootstrap case, decisions about the ranges and values of the parameters were taken. The main idea is to select an optimal combination of parameters for our datasets in the study. In conclusion, the final values for the bootstrap case are: **bootstrap = True; size of bootstrap = 1; number of trees = 50; max depth = 1; min leaf = 0.13; max features = 1.**

Appendix Tables

Across this section there are tables that supplement the hyperparameter study.

Table 1.3: Combinations and accumulated NRF results of the first DOE for the non bootstrap case.

Combination	Nº of Trees	Max Depth	Max Features	Min Leaf	NRF
1	10	0.100	0.250	0.010	390
2	50	0.100	0.250	0.010	329
3	10	1.000	0.250	0.010	379
4	50	1.000	0.250	0.010	374
5	10	0.100	1.000	0.010	375
6	50	0.100	1.000	0.010	384
7	10	1.000	1.000	0.010	372
8	50	1.000	1.000	0.010	400
9	10	0.100	0.250	0.250	402
10	50	0.100	0.250	0.250	427
11	10	1.000	0.250	0.250	368
12	50	1.000	0.250	0.250	402
13	10	0.100	1.000	0.250	418
14	50	0.100	1.000	0.250	451
15	10	1.000	1.000	0.250	439
16	50	1.000	1.000	0.250	455
17 - C	30	0.550	0.625	0.130	423
18 - C	30	0.550	0.625	0.130	432
19 - C	30	0.550	0.625	0.130	419
20 -C	30	0.550	0.625	0.130	406

Table 1.4: Summary of the *ANOVA* results of the first DOE for the non bootstrap case.

Factor	Effect	Std.Err	t	p-value
(1)MinLeaf	44.8750	10.67008	4.20568	**0.002287**
(2)Max Features	27.8750	10.67008	2.61244	**0.028158**
(3)Max Depth	1.6250	10.67008	0.15229	0.882314
(4)Number of Trees	9.8750	10.67008	0.92548	0.378861
1 by 2	13.1250	10.67008	1.23007	0.249852
1 by 3	-10.1250	10.67008	-0.94891	0.367447
1 by 4	17.1250	10.67008	1.60495	0.142967
2 by 3	7.8750	10.67008	0.73804	0.479288
2 by 4	11.6250	10.67008	1.08949	0.304246
3 by 4	8.3750	10.67008	0.78490	0.452674

Table 1.5: Combinations and accumulated NRF results of the second DOE for the non bootstrap case.

Nº of Trees	Max Features	Min Leaf	NRF
10	0.25	0.13	388
50	0.25	0.13	415
10	1.00	0.13	442
50	1.00	0.13	404
10	0.63	0.01	357
50	0.63	0.01	434
10	0.63	0.25	414
50	0.63	0.25	439
30	0.25	0.01	413
30	1.00	0.01	359
30	0.25	0.25	360
30	1.00	0.25	448
30	0.63	0.13	400
30	0.63	0.13	411
30	0.63	0.13	396

Table 1.6: Combinations and accumulated NRF results of the first DOE for the bootstrap case.

Combination	Nº of Trees	Size of the Bootstrap	Min Leaf	Max Depth	Max Features	NRF
1	50	0.600	0.010	0.100	1.000	338
2	100	0.600	0.010	0.100	0.250	430
3	50	1.000	0.010	0.100	0.250	379
4	100	1.000	0.010	0.100	1.000	359
5	50	0.600	0.250	0.100	0.250	522
6	100	0.600	0.250	0.100	1.000	492
7	50	1.000	0.250	0.100	1.000	380
8	100	1.000	0.250	0.100	0.250	402
9	50	0.600	0.010	1.000	0.250	407
10	100	0.600	0.010	1.000	1.000	410
11	50	1.000	0.010	1.000	1.000	350
12	100	1.000	0.010	1.000	0.250	377
13	50	0.600	0.250	1.000	1.000	499
14	100	0.600	0.250	1.000	0.250	491
15	50	1.000	0.250	1.000	0.250	400
16	100	1.000	0.250	1.000	1.000	387
17 - C	75	0.800	0.130	0.550	0.625	402
18 - C	75	0.800	0.130	0.550	0.625	386
19 - C	75	0.800	0.130	0.550	0.625	371
20 - C	75	0.800	0.130	0.550	0.625	402

Table 1.7: Summary of the *ANOVA* results of the first DOE for the bootstrap case.

Factor	Effect	Std.Err	t	p-value
(1)Max Features	-24.1250	12.40026	-1.94552	0.123591
(2)Max Depth	2.3750	12.40026	0.19153	0.857441
(3)MinLeaf	65.3750	12.40026	5.27207	**0.006203**
(4)Size of the bootstrap	-69.3750	12.40026	-5.59464	**0.005009**
(5)Number of trees	9.1250	12.40026	0.73587	0.502630
1 by 2	16.8750	12.40026	1.36086	0.245185
1 by 3	9.8750	12.40026	0.79635	0.470417
1 by 4	3.6250	12.40026	0.29233	0.784568
1 by 5	11.1250	12.40026	0.89716	0.420351
2 by 3	-7.1250	12.40026	-0.57458	0.596342
2 by 4	-3.8750	12.40026	-0.31249	0.770279
2 by 5	-6.8750	12.40026	-0.55442	0.608825
3 by 4	-39.3750	12.40026	-3.17534	**0.033685**
3 by 5	-16.3750	12.40026	-1.32054	0.257144
4 by 5	-5.1250	12.40026	-0.41330	0.700585

Table 1.8: Combinations and accumulated NRF results of the second DOE for the bootstrap case.

Size of the Bootstrap	Max Features	Min Leaf	NRF
0.6	0.625	0.010	404
1.0	0.625	0.010	402
0.6	0.625	0.250	491
1.0	0.625	0.250	364
0.6	0.250	0.130	446
1.0	0.250	0.130	401
0.6	1.000	0.130	367
1.0	1.000	0.130	360
0.8	0.250	0.010	436
0.8	0.250	0.250	410
0.8	1.000	0.010	394
0.8	1.000	0.250	416
0.8	0.625	0.130	376
0.8	0.625	0.130	383
0.8	0.625	0.130	394
