# Supplementary material for: PhyloMissForest: a random forest framework to construct phylogenetic trees with missing data
Source: BMC Genomics. 2022 May 18;23:377. doi: 10.1186/s12864-022-08540-6 (PMC9116704; doi:10.1186/s12864-022-08540-6)
Supplement: Supplementary file 3 — Additional file 3 Imputation with Random Forest. An example on the use of random forests for phylogenetic data imputation. [file 12864_2022_8540_MOESM3_ESM.pdf]

Imputation with Random Forest - Example

In order to better understand the behaviour of the presented algorithm, we introduce an example of the decisions performed during the imputation process.

As it is shown in Figure 1, given an input matrix M, the first step of the algorithm is to create the boolean mask of the inputted matrix.

	OTU 1	OTU 2	OTU 3	OTU 4	OTU 5
**OTU 1**	0	-	-	10	-
**OTU 2**	-	0	-	9	8
**OTU 3**	-	-	0	4	5
**OTU 4**	10	9	4	0	3
**OTU 5**	-	8	5	3	0

	OTU 1	OTU 2	OTU 3	OTU 4	OTU 5
**OTU 1**	False	True	True	False	True
**OTU 2**	True	False	True	False	False
**OTU 3**	True	True	False	False	False
**OTU 4**	False	False	False	False	False
**OTU 5**	True	False	False	False	False

Figure 1: Step of creating the boolean mask, where '-' represents a missing value.

The mask created by the algorithm is used to locate the original missing values at each cycle of the imputation process. Moving to the initialization step of the algorithm, the missing values can be initially guessed by calculating the mean of each column. This process is represented in Figure 2.

	Mean
**OTU 1**	5
**OTU 2**	5.667
**OTU 3**	3
**OTU 4**	5.2
**OTU 5**	4

	OTU 1	OTU 2	OTU 3	OTU 4	OTU 5
**OTU 1**	0	5.667	3	10	4
**OTU 2**	5	0	3	9	8
**OTU 3**	5	5.667	0	4	5
**OTU 4**	10	9	4	0	3
**OTU 5**	5	8	5	3	0

Figure 2: Initial guess of the missing values.

As shown in Figure 2, on the left, there is a vector with the mean of the known values for each column. On the right, the missing values of Figure 1 are replaced by the mean of the known values for each column. Once concluded the initialization process, the imputation loop starts. Firstly, the algorithm will decide the order of the columns to follow during the imputation cycle. In order to accomplish this, the number of missing values per column is calculated and the columns will be sorted, starting with the column with less missing values to the column with the highest number of missing values. This procedure is depicted in Figure 3.

	OTU 1	OTU 2	OTU 3	OTU 4	OTU 5
**OTU 1**	False	True	True	False	True
**OTU 2**	True	False	True	False	False
**OTU 3**	True	True	False	False	False
**OTU 4**	False	False	False	False	False
**OTU 5**	True	False	False	False	False

	Missing Values	Order
**OTU 1**	3	**OTU 4**
**OTU 2**	2	**OTU 5**
**OTU 3**	2	**OTU 2**
**OTU 4**	0	**OTU 3**
**OTU 5**	1	**OTU 1**

Figure 3: Definition of the imputation order.

In the example of Figure 3, aiming to know how many missing values each OTU has, the algorithm counts the number of true occurrences per column in the mask. Having the referred number, the imputa-

tion order occurs from the column with less missing values to the one with the largest number of missing occurrences. As OTU 4 has no missing value, this is the first OTU in the vector of the imputation order. OTU 5 comes in second place, while OTUs 2 and 3 come in the third and fourth place, respectively, since both have the same number of missing values, and therefore, the order is defined by the lowest index. Finally, it comes OTU 1 that is the one with the largest number of missing values.

Once defined the order of the columns in which the imputation will occur, the algorithm starts a loop to iterate column by column, following the order previously defined. In this example, it will identify that the first OTU (OTU 4) has no missing values, so it will proceed immediately with the next OTU. Knowing that OTU 5 has one missing value, the algorithm, with the help of the mask, will divide the dataset into 3 parts: X_{obs}, X_{miss} and y_{obs}. In Figure 4, there is an example of the process of splitting the dataset.

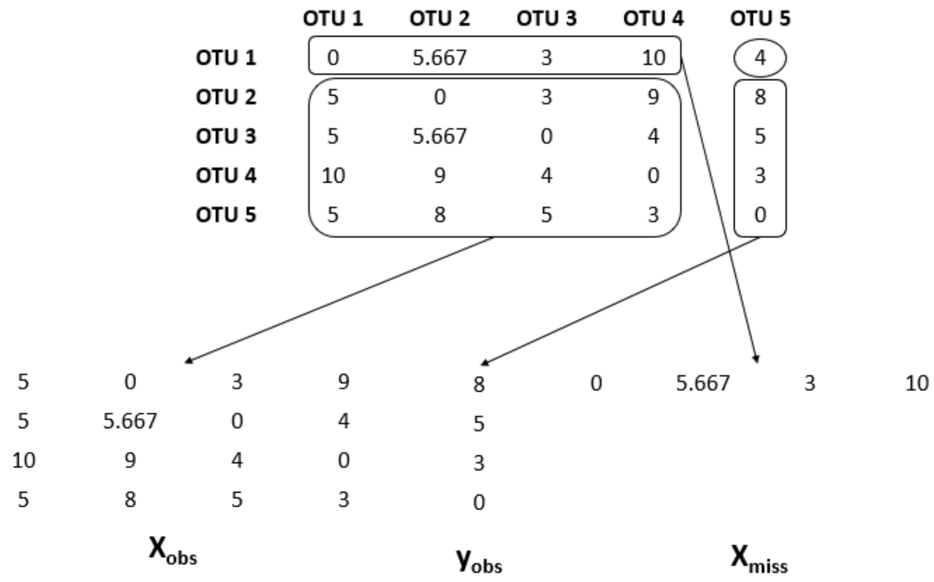

Figure 4: Splitting the dataset in order to prepare the random forest train.

Examining Figure 4 and taking as a reference the column that is being imputed, in this case OTU 5:

- X_{obs} is the part of the dataset composed by the values of the other OTUs, that correspond to the known values of the OTU that is being imputed;
- y_{obs} is the known values of the OTU that is being imputed;
- X_{miss} is the part of the dataset composed by all the values of the other OTUs, where the values of the OTU that is being imputed are unknown;

Finished the procedure to split the dataset, the algorithm trains a random forest with X_{obs} and y_{obs}. When the training process finishes, the prediction function of the random forest is executed, giving as an input the X_{miss}, and the y_{miss} is returned by the referred function. Recalling the example from the previous steps, Figure 5 shows the process of imputing the values returned from the prediction of the forest in the distance matrix. In this example, the random forest returns a prediction of 5.8 for the missing cell. This value replaces the initial guess of the missing cell.

	OTU 1	OTU 2	OTU 3	OTU 4	OTU 5
**OTU 1**	0	5.667	3	10	5.8
**OTU 2**	5	0	3	9	8
**OTU 3**	5	5.667	0	4	5
**OTU 4**	10	9	4	0	3
**OTU 5**	5	8	5	3	0

5.8
$\mathbf{y}_{miss}$

Figure 5: Imputing the values returned from the prediction of the forest in the matrix.

Once the imputation of OTU 5 is finished, the algorithm moves to the next column in the vector of the imputation order. When all the missing values are imputed, that is, when all the columns have been processed by the iterative loop, the error between the old matrix and the new one is calculated. If the new error is lower then the old, another iteration is performed, otherwise, the old matrix is returned and the imputation process is over.
